# Universal Antibody‐Engineered Lipid Nanoparticles Potentiate Chemo‐Immunotherapy Against Triple‐Negative Breast Cancer by Reprogramming Tumor Cell Metabolism

**DOI:** 10.1002/advs.202518468

**Published:** 2026-01-27

**Authors:** Yeneng Dai, Jiaqi Wang, Yu Liu, Guanda Jiao, Yuheng Gu, Yang Liu, Shengyu Fu, Xing Fan, Jialin Li, Ziang Guo, Kam Tong Leung, Lipeng Zhu, Qi Zhao

**Affiliations:** ^1^ Cancer Centre Institute of Translational Medicine Faculty of Health Sciences University of Macau Taipa Macau SAR China; ^2^ MoE Frontiers Science Center for Precision Oncology University of Macau Taipa Macau SAR China; ^3^ School of Life Sciences Central South University Changsha China; ^4^ Department of Paediatrics the Chinese University of Hong Kong Hong Kong China

**Keywords:** chemo‐immunotherapy, lipid nanoparticles, microenvironment remodeling, mitochondria metabolism, ROR1 antibody

## Abstract

Despite the promise of combining chemotherapy and immunotherapy for triple‐negative breast cancer (TNBC), challenges remain due to targeting deficiencies and accelerated T cell exhaustion within the immunosuppressive tumor microenvironment (TME). To address these limitations, we developed a drug‐encapsulated lipid nanoparticle (LNP) system to reprogram tumor metabolism and reverse acquired immune tolerance. This LNP encapsulates the cytotoxic agent monomethyl auristatin E (MA) and the immunomodulator metformin (Met), followed by conjugated to a human receptor tyrosine kinase‐like orphan receptor 1 antibody (ROR1 Ab) via an acid‐labile linker. ROR1 Ab‐mediated active targeting enables precise tumor localization through NIR‐II fluorescence imaging, and enhances chemotherapeutic efficacy while minimizing off‐target toxicity. Critically, metformin incorporated into the lipid formulation inhibits both membrane and cytoplasmic PD‐L1 expression and reduces TGF‐β1 levels by suppressing mitochondrial oxidative phosphorylation (OXPHOS), thereby restoring T lymphocyte activity and amplifying the immune response. This work demonstrates a novel approach to enhance TNBC treatment through targeted modulation of tumor metabolism and immune activation.

## Graphical abstract

A universal ROR1 antibody‐engineered lipid nanoparticle (LNP) is constructed by sequential drug encapsulation and antibody conjugation. The LNP not only enables enhanced chemotherapeutic efficiency by targeting drug delivery and precise localization of deep‐seated tumors through NIR‐II fluorescence imaging, but also remodels immunosuppressive TME through metabolism reprogramming, thereby potentiating the infiltration and immune activity of T lymphocytes against lung cancer tumors.

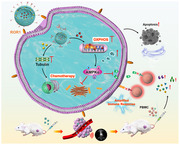

## 1 Introduction

Triple‐negative breast cancer (TNBC) has been regarded as the most malignant subtype of breast cancer. Chemotherapy can induce better responses of TNBC compared with surgery and radiotherapy [1]. However, long‐term systemic distribution of chemotherapeutic agents may lead to nonspecific toxic side effects to normal tissues due to the lack of targeted receptors in TNBC, seriously influencing the quality of life of TNBC patients [2, 3]. Therefore, imaging‐mediated real‐time visualization of the in vivo drug distribution is important for subsequent precise intervention. In addition, a single chemotherapy modality cannot completely eradicate tumor cells, easily provoking tumor metastasis and recurrence. Consequently, it is desired to develop a targeted therapeutic strategy in combination with other treatment modalities toward TNBC [4]. Receptor tyrosine kinase‐like orphan receptor 1 (ROR1), a member of the ROR family with tyrosine kinase domains that bind ligands, is overexpressed on the membrane surface of various cancer cells, including ovarian cancer and TNBC [5, 6]. It has been established that ROR1 contributes to activating noncanonical WNT signaling to induce breast cancer proliferation and metastasis, making it a potential candidate for targeted theranostics of TNBC [7, 8]. Recently, fluorescence imaging in the second near‐infrared (NIR‐II, 1000–1700 nm) window has attracted considerable attention due to the deeper light penetration depth and reduced tissue autofluorescence [9, 10], which not only enables real‐time monitoring of drug distribution, but also locates deep‐seated tumors, providing key information for precise tumor treatment [11, 12].

Immunotherapy has emerged as a promising therapeutic modality for tumor elimination in combination with chemotherapy [13, 14]. Unfortunately, the immune output is still obstructed by immunosuppressive tumor microenvironment (TME) [15, 16, 17]. The overexpressed programmed cell death ligand 1 (PD‐L1) on the surface of tumor cells can induce immune escape by binding to programmed cell death protein 1 (PD‐1) on the surface of T lymphocytes [18]. Immune checkpoint blockade therapy targeting the PD‐1/PD‐L1 axis has become a mainstream strategy to enhance immune response in clinical practice [19, 20]. However, these blocking antibodies may evoke systemic immunogenicity risk due to nonspecific accumulation, resulting in suboptimal immune efficacy [21]. Moreover, they can only block the recognition between the tumor cell membrane‐located PD‐L1 and T cells without affecting the expression of cytoplasm‐located PD‐L1, which can be transferred to the cell membrane to weaken the immune response [22, 23]. More importantly, the immunosuppressive factor secreted from tumor cells, transforming growth factor‐β1 (TGF‐β1), can further weaken the immune activity of T cells along with a hypoxic microenvironment, causing sustained T cell exhaustion [24, 25]. Therefore, there is an urgent need to develop a more effective PD‐L1 inhibition mechanism along with TGF‐β1 depression and hypoxia relief, thereby reactivating T cells to amplify the immune response.

Recent studies have shown that most cancer cells rely on mitochondria‐mediated oxidative phosphorylation (OXPHOS) to generate adenosine triphosphate (ATP) by consuming oxygen [26]. OXPHOS inhibition can promote the phosphorylation of AMP‐activated protein kinase (p‐AMPK), leading to phosphorylation of the S195 site of PD‐L1 [27]. This will induce abnormal glycosylation, endoplasmic reticulum (ER) accumulation, and degradation of PD‐L1, ultimately reducing the expression of PD‐L1 in the cell membrane and cytoplasm [28]. Moreover, the secretion of TGF‐β1 could also be obstructed by mitochondrial AMPK phosphorylation [29, 30]. Therefore, mitochondrial metabolism chaos has become a new target for enhancing immune activation through OXPHOS inhibition.

**SCHEME 1:**
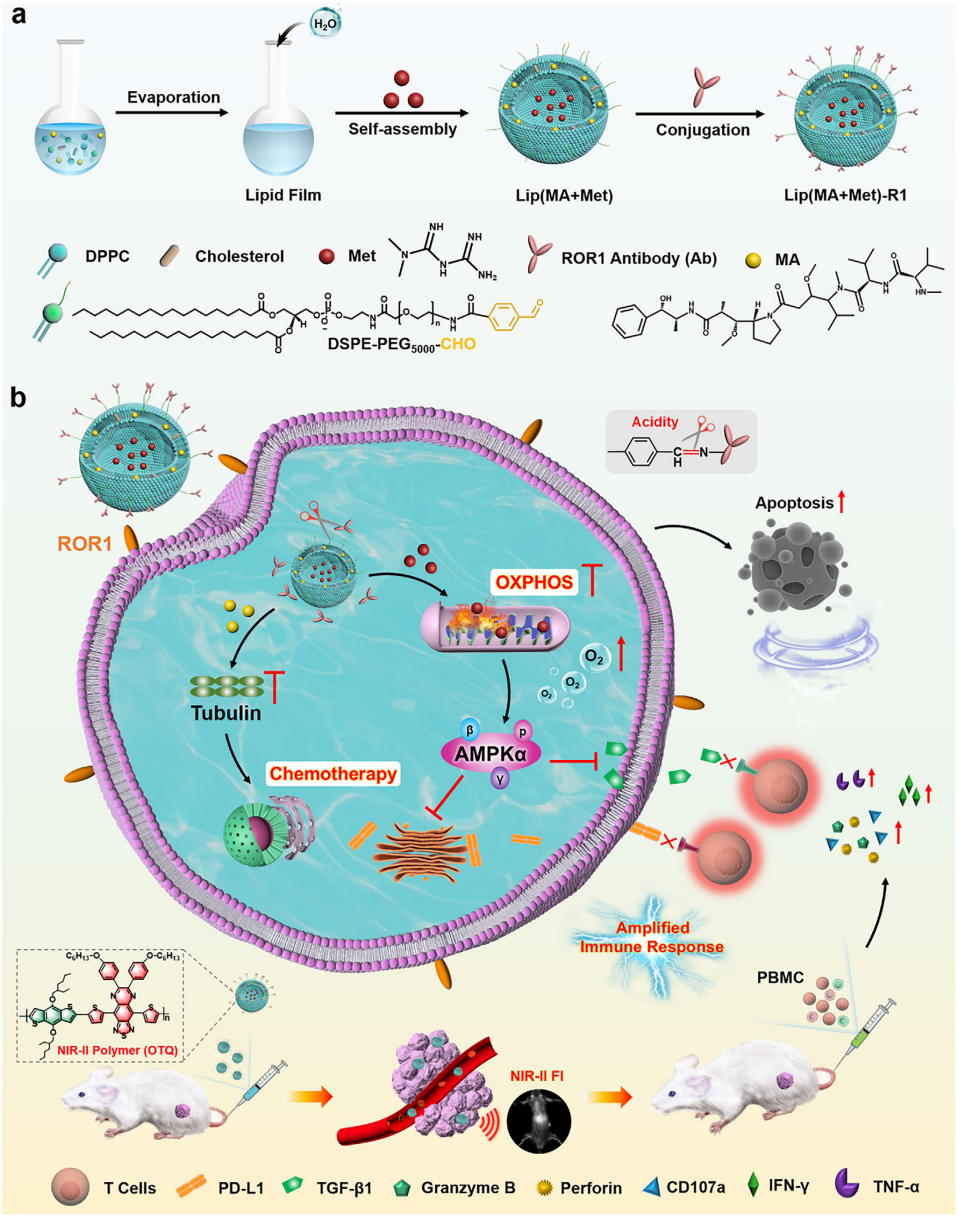
Schematic diagram of Lip(MA+Met)‐R1 for potentiating chemo‐immunotherapy via tumor metabolism reprogramming. (a) Preparation of ROR1 Ab‐engineered lipid nanoparticle Lip(MA+Met)‐R1. (b) NIR‐II fluorescence imaging‐guided amplified chemo‐immunotherapy against TNBC by mitochondrial metabolism regulation.

To solve the abovementioned challenges, herein, we designed and prepared a targeted antibody‐engineered lipid nanoparticle (LNP) Lip(MA+Met)‐R1 with mitochondrial metabolism reprogramming capability by encapsulating the chemotherapy drug monomethyl auristatin E (MA) and the immunomodulator metformin (Met) into liposomes, followed by conjugation with human ROR1 antibody (Ab) through an acid‐labile linker (Scheme 1). By doping with NIR‐II fluorescence probes, the LNP enables precise localization of TNBC tumors by NIR‐II fluorescence imaging. After ROR1 Ab mediated active targeting to TNBC cells through the specific binding of the antibody to the receptor, the LNP is internalized into the lysosome of tumor cells, where the acidic condition triggers the detachment of liposomes from ROR1 Ab, releasing the payload into the cytoplasm. The cytotoxin MA induces tumor cell apoptosis through the functional inhibition of tubulin. Moreover, Met serves as a mitochondrial OXPHOS inhibitor to reverse the immunosuppressive TME through the inhibition of membrane and intracellular PD‐L1 together with TGF‐β1 depression, restoring the immune activity of T lymphocytes and improving the anti‐tumor killing effect in combination with chemotherapy. This work integrates chemotherapy and tumor metabolism regulation into a single nanoplatform engineered with a targeted antibody, providing a new strategy to amplify the efficiency of chemo‐immunotherapy against TNBC.

## 2 Results and Discussion

### 2.1 Preparation and Characterization of Antibody‐Engineered LNPs

**FIGURE 1:**
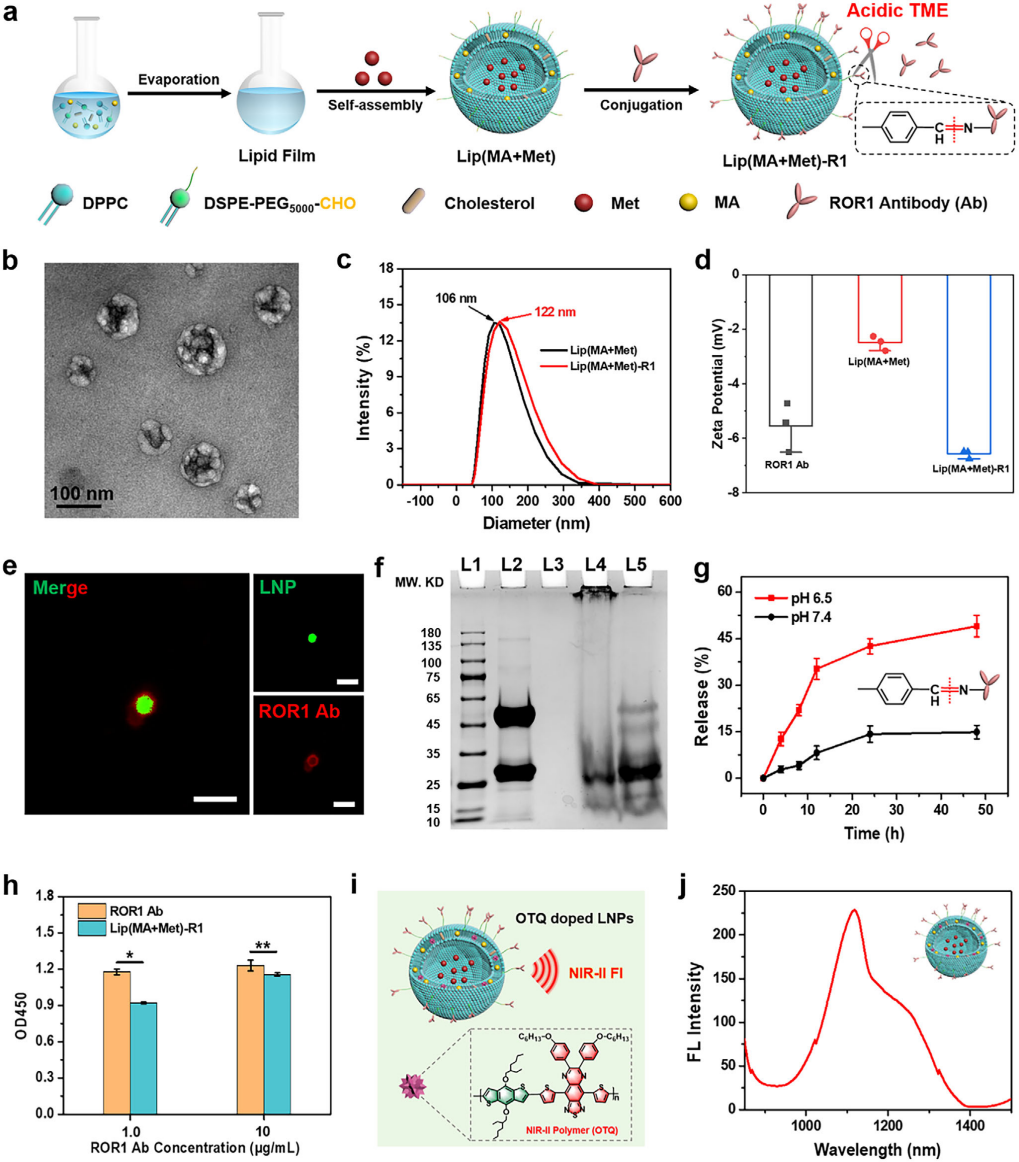
Preparation and characterization of ROR1 Ab conjugated LNPs. (a) Schematic diagram of the preparation of Lip(MA+Met)‐R1. (b) Transmission electron microscopy (TEM) image of Lip(MA+Met)‐R1. (c) Hydrodynamic diameters of different samples. (d) Zeta potential of different components. (e) Fluorescence colocalization analysis of ROR1 Ab and the liposome after doped with RhB. Scale bar: 200 nm. (f) SDS‐PAGE analysis of ROR1 Ab and Lip(MA+Met)‐R1 with or without acidic conditions. L1: Marker, L2: ROR1 Ab, L3: Lip(MA+Met), L4: Lip(MA+Met)‐R1 + pH 7.4, L5: Lip(MA+Met) + pH 6.5. (g) Cumulative release of ROR1 Ab from Lip(MA+Met)‐R1 at different pH conditions. (h) The binding activity of ROR1 Ab and Lip(MA+Met)‐R1 toward ROR1 antigen (n = 3 per group). (i) Schematic illustration of LNPs doped with NIR‐II polymer OTQ. (j) The fluorescence emission spectrum of Lip(MA+Met)‐R1 doped with NIR‐II polymer OTQ. ^*^ *p* < 0.05, ^**^ *p* < 0.01.

ROR1 has been proven to be overexpressed in TNBC, which has become a therapeutic target of antibody‐drug conjugates (ADC). To prepare antibody‐conjugated LNPs, aldehyde‐modified lipid components (DSPE‐PEG_5000_‐CHO) were first synthesized via amidation reaction (Scheme S1), and their structure was confirmed by proton nuclear magnetic resonance (^1^H NMR) spectroscopy (Figure S1). Targeted antibody‐conjugated LNPs were prepared by a thin film hydration method based on self‐assembly of lipid components and payloads, followed by modification with ROR1 Ab via reversible hydrazone bond, in which water‐soluble immunomodulator Met served as the core and hydrophobic chemotherapeutic drug MA was embedded in the lipid bilayer (Figure 1a). Transmission electron microscopy images showed uniformly dispersed spherical morphologies of Lip(MA+Met) and Lip(MA+Met)‐R1 (Figure 1b and Figure S3). After conjugation with ROR1 Ab, the size of the LNPs increased from 106 nm of Lip(MA+Met) to 122 nm by dynamic light scattering analysis (Figure 1c). The surface zeta potential of ROR1 Ab functionalized nanoparticles decreased from −2.79 to −6.5 mV, attributed to the negative charge of ROR1 Ab (Figure 1d). To visualize the conjugation of the antibody and the LNP, the fluorescent dye rhodamine B (RhB)‐doped Lip(MA+Met)‐R1 was incubated with a fluorescence‐labeled secondary antibody. Confocal laser scanning microscopy (CLSM) images demonstrated that the ROR1 Ab with red fluorescence was uniformly dispersed on the spherical surface of LNPs with green fluorescence (Figure 1e), further verifying the successful conjugation of ROR1 Ab to the LNP. In addition, compared with free ROR1 Ab, the significant protein retardation of Lip(MA+Met)‐R1 lane at pH 7.4 ascertained the successful conjugation of antibody on the nanomedicine by sodium dodecyl sulfate‐polyacrylamide gel electrophoresis (SDS‐PAGE) (Figure 1f). An obvious band shift of ROR1 Ab was observed in Lip(MA+Met)‐R1 under acidic conditions (pH 6.5), demonstrating the separation of the antibody and liposomes. This is ascribed to the dissociation of the acid‐labile linker (hydrazone bond), which is beneficial for the cleavage and release of the antibody‐LNP conjugates within the lysosomes of the TME. As shown in Figure 1g, Lip(MA+Met)‐R1 exhibited a significant release of ROR1 Ab under slightly acidic conditions compared with physiological pH. Moreover, stronger acidity can further facilitate the dissociation of ROR1 Ab from LNPs compared with that at pH 6.5 (Figure S4), which is beneficial for the separation of ADCs in the endo‐lysosomal pathway with acidic conditions. To evaluate the expression level of ROR1 on MDA‐MB‐231 cells, ROR1 antibody was incubated with different cancer cell lines, and flow cytometry analysis revealed the specific presence of ROR1 antigen on the surface of the MDA‐MB‐231 cells compared with other cancer cells (Figure S5). Compared with ROR1 Ab, the binding activity of Lip(MA+Met)‐R1 toward ROR1 antigen was almost unchanged (Figure 1h). The kinetic analysis showed that Lip(MA+Met)‐R1 maintained a binding pattern similar to that of ROR1 Ab, with a K_D_ of 14.0 nm (Figure S6). In the cell binding assay, Lip(MA+Met)‐R1 showed the prominent surface binding ability toward MDA‐MB‐231 cells (Figure S7). These results demonstrate that Lip(MA+Met)‐R1 possesses a positively active targeting ability to MDA‐MB‐231 cells overexpressing ROR1 antigen.

The NIR‐II semiconducting polymer (OT‐TTQ, OTQ) was synthesized by Still polymerization using strong electron‐donating (4,8‐bis((2‐ethylhexyl)oxy)benzo[1,2‐b:4,5‐b’]dithiophene‐2,6‐diyl)bis(trimethylstannane) (OT) as the donor unit and strong electron‐withdrawing 6,7‐Bis(4‐(hexyloxy)phenyl)‐4,9‐di(thiophen‐2‐yl)‐ [1, 2, 3, 7, 8]thiadiazolo [3, 4‐g]quinoxaline (TTQ) as the acceptor unit (Scheme S2), whose chemical structure was characterized by ^1^H NMR spectroscopy (Figure S2). The narrow energy band gap between HOMO and LUMO indicated the possible long‐wavelength absorption capability by the density functional theory (DFT) calculation (Figure S8a, b), evidenced by the broad absorption band from 400 to 1100 nm with an absorption peak at 850 nm (Figure S8c). OTQ exhibited significant NIR‐II fluorescence emission under 808 nm laser excitation, with a bright NIR‐II fluorescence image (Figure S8d). OTQ was doped into LNPs by the thin film hydration method (Figure 1i), showing a spherical morphology with a diameter of approximately 122 nm and similar light absorption capacity as free OTQ (Figures S9 and S10). OTQ‐doped LNPs exhibited prominent NIR‐II fluorescence emission from 1000 to 1400 nm, with an emission peak of about 1120 nm (Figure 1j), which could serve as a targeted diagnosis agent for precise localization of deep‐seated tumors.

### 2.2 Cellular Uptake and 3D Tumor Spheroid Penetration Analysis

**FIGURE 2:**
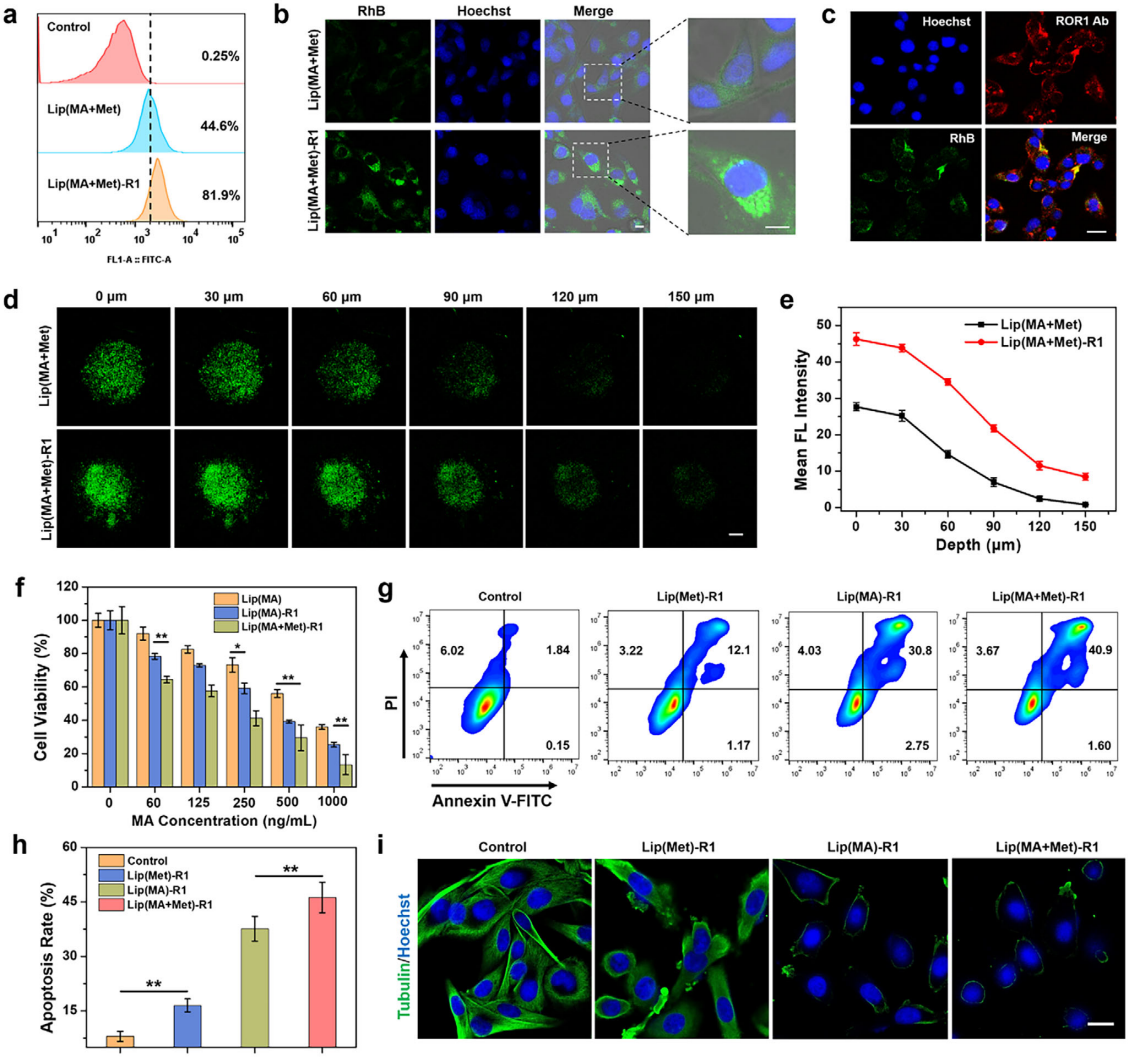
Cellular uptake, cytotoxicity, and tubulin inhibition analysis. (a) Flow cytometric analysis and (b) fluorescence images of cellular uptake in MDA‐MB‐231 cells. Scale bar: 10 µm. (c) Fluorescence colocalization images of RhB‐doped Lip(MA+Met)‐R1 and ROR1 Ab in MDA‐MB‐231 cells. Scale bar: 20 µm. (d) Z‐stack confocal microscopy images and (e) fluorescence intensity analysis at different depths in 3D tumor spheroids treated with Lip(MA+Met) and Lip(MA+Met)‐R1. Scale bar: 200 µm. (f) Cell viability of MDA‐MB‐231 cells after various treatments (n = 5 per group). (g) Flow cytometric analysis and (h) statistical data of cell apoptosis after various treatments (n = 3 per group). (i) Fluorescence images of tubulin expression after different treatments. Scale bar: 20 µm. ^*^ *p* < 0.05, ^**^ *p* < 0.01.

To investigate the targeted binding capability of ROR1 Ab toward TNBC, MDA‐MB‐231 cells were incubated with RhB‐doped Lip(MA+Met) and Lip(MA+Met)‐R1, respectively. Lip(MA+Met)‐R1 treated cells exhibited much higher RhB fluorescence than those treated with Lip(MA+Met), with an uptake ratio of approximately 81.9% at 24 h by flow cytometry analysis (Figure 2a), indicating ROR1 Ab mediated higher cellular binding. CLSM images revealed the brighter fluorescence in Lip(MA+Met)‐R1 treated tumor cells after 12 h incubation (Figure 2b), ascribed to the active binding of ROR1 Ab toward ROR1 antigen on the surface of MDA‐MB‐231 cells. To further visualize the binding efficiency of ROR1 Ab against tumor cells, MDA‐MB‐231 cells were incubated with ROR1 Ab and Lip(MA+Met)‐R1, respectively, followed by labeling with the secondary antibodies with fluorophores. The ROR1 Ab was clearly observed in the cell membrane and cytoplasm by red fluorescence, and the tumor cells treated with Lip(MA+Met)‐R1 showed a fluorescence colocalization of ROR1 Ab and LNPs (Figure 2c and Figure S11).

Lysosome escape behaviors of Lip(MA+Met)‐R1 were investigated according to previous reports [31, 32, 33]. As shown in Figure S12a, low fluorescence overlap between RhB and the lysosomal tracker was observed after 4 h of incubation, indicating that Lip(MA+Met)‐R1 had not yet been completely captured by lysosomes. At 12 h, tumor cells exhibited significant fluorescence co‐localization with a Pearson's correlation coefficient (PPC) value of 0.84 (Figure S12b), verifying the lysosomal endocytosis. After prolonging to 16 h, the PPC value dramatically decreased to 0.41, indicating remarkable lysosomal escape. These results confirm the excellent lysosomal escape capability of Lip(MA+Met)‐R1 for efficient cellular uptake. After binding to the ROR1 antigen of tumor cells, Lip(MA+Met)‐R1 was internalized into endosomes and lysosomes and then further dispersed into the cytoplasm after lysosome escape [5]. In addition, ROR1 Ab‐mediated tumor cell spheroid infiltration efficiency was further evaluated. The penetration depth of Lip(MA+Met)‐R1 in 3D tumor spheroids was deeper than that of Lip(MA+Met), verified by higher fluorescence at different depths of the cell spheroids (Figure 2d). Even at the depth of 150 µm, the fluorescence intensity of the cell spheroid treated with Lip(MA+Met)‐R1 was 10.15 fold higher than that of the Lip(MA+Met) treated group (Figure 2e). These results confirm that ROR1 Ab conjugation could significantly enhance the infiltration and penetration of LNPs in tumor spheroids via antibody‐mediated targeted cellular uptake.

### 2.3 Cytotoxicity and Tubulin Inhibition Analysis

To evaluate the combination therapeutic effect of Lip(MA+Met)‐R1 against TNBC, the cytotoxicity of lipid nanomedicines encapsulating tubulin inhibitor MA alone was first evaluated. As shown in Figure 2f, Lip(MA)‐R1 showed MA concentration‐dependent chemotherapeutic effects toward MDA‐MB‐231 cells, with a lower cell viability at low MA concentrations compared with Lip(MA). This is attributed to ROR1 Ab‐mediated targeted delivery and improved tubulin inhibition. In addition, the anti‐tumor activity of mitochondrial respiration inhibitor Met was also investigated. Lip(Met)‐R1 showed a certain tumor inhibitory efficiency with increased Met concentrations (Figure S13). The tumor cell killing rate could reach approximately 34.75% at a high concentration of 200 µg/mL, which may be ascribed to the disruption of mitochondrial function through Met‐mediated oxidative phosphorylation (OXPHOS) inhibition. Particularly, Lip(MA+Met)‐R1 exhibited the highest cytotoxicity, verified by the lowest cell viability of about 13.29% at the high concentration, due to the combination effects of chemotherapy and mitochondrial metabolism regulation. The anti‐tumor killing efficacy was further evidenced by the obvious shrinking morphology of apoptotic cells (Figure S14). The quantitative analysis by flow cytometry revealed the highest apoptotic ratio in the Lip(MA+Met)‐R1 treated group (Figure 2g, h). In particular, the mechanism of MA‐mediated chemotherapy was further explored by tubulin expression analysis after various treatments. Compared with other groups, less green fluorescence was observed in the Lip(MA)‐R1 and Lip(MA+Met)‐R1 treated groups, indicating reduced tubulin expression in tumor cells (Figure 2i). ROR1 Ab‐modified MA LNPs could inhibit tubulin polymerization and destroy the microtubule network, thereby inhibiting cell division and proliferation, ultimately leading to cell death. These results demonstrate that LNPs possess excellent anti‐tumor cell killing effects toward MDA‐MB‐231 cells through the combination therapeutic effects of chemotherapy and metabolic regulation.

### 2.4 In Vitro Mitochondrial Dysfunction, AMPK Activation, PD‐L1, and TGF‐β1 Depression

**FIGURE 3:**
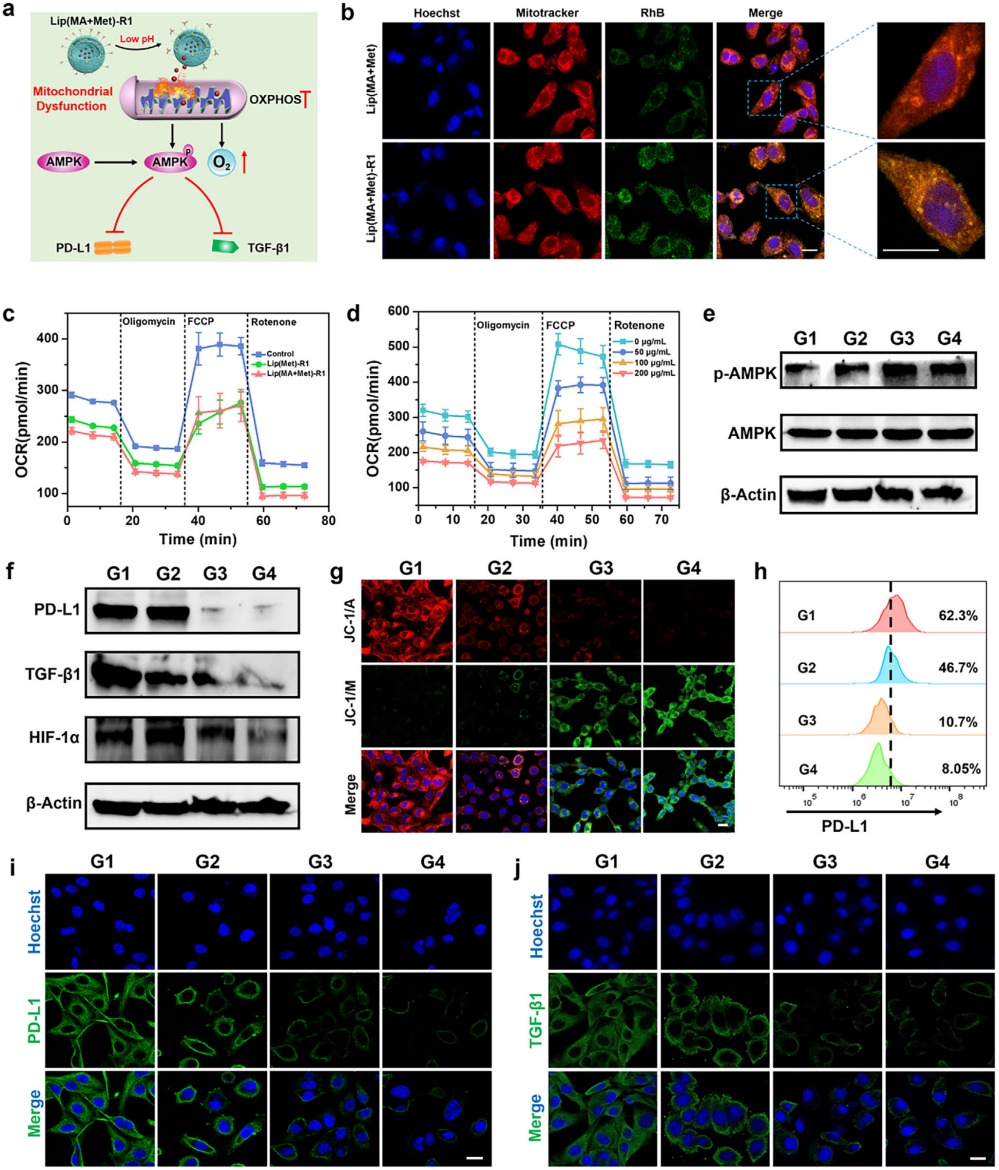
The mitochondrial dysfunction, AMPK activation, and the inhibition of PD‐L1 and TGF‐β1. (a) Schematic diagram of Lip(MA+Met)‐R1 mediated mitochondrial metabolism reprogramming. (b) The fluorescence localization images of Lip(MA+Met) and Lip(MA+Met)‐R1 in mitochondria. Scale bar: 20 µm. (c) The mitochondrial OCR of MDA‐MB‐231 cells with different treatments (n = 3 per group). (d) The mitochondrial OCR of MDA‐MB‐231 cells treated with Lip(MA+Met)‐R1 with various Met concentrations (n = 3 per group). (e) The expression of p‐AMPK protein by western blot analysis in MDA‐MB‐231 cells after various treatments. (f) The western blot analysis of PD‐L1, TGF‐β1, and HIF‐1α levels in MDA‐MB‐231 subjected to various treatments. (g) MMP detection of MDA‐MB‐231 cells with various treatments by JC‐1 probes. Scale bar: 20 µm. (h) Flow cytometric analysis of membrane‐located PD‐L1 level in MDA‐MB‐231 cells. Representative immunofluorescence images of the expression of (i) PD‐L1 and (j) TGF‐β1 in MDA‐MB‐231 cells. Scale bar: 20 µm. G1: Control, G2: Lip(MA)‐R1, G3: Lip(Met)‐R1, G4: Lip(MA+Met)‐R1.

To investigate the influence of LNPs on mitochondrial function of tumor cells, the fluorescence localization of intracellular mitochondria and LNPs in MDA‐MB‐231 cells was first detected by CLSM utilizing a mitochondrial probe. Lip(MA+Met)‐R1 treated cells exhibited more green fluorescence of LNPs, overlapped with red fluorescence representing mitochondria (Figure 3b), indicating that Lip(MA+Met)‐R1 could be effectively internalized into tumor cells and transported to mitochondria through ROR1 Ab mediated active targeting. Oxygen consumption rate (OCR) of MDA‐MB‐231 cells was evaluated after various treatments. The basal respiration, ATP‐linked respiration, and maximum respiration of tumor cells were measured after injection with oligomycin, trifluoromethoxy carbonylcyanide phenylhydrazone (FCCP), and rotenone/antimycin A. Both Lip(MA+Met)‐R1 and Lip(Met)‐R1 exhibited significant respiration inhibition and OCR reduction compared with the control group (Figure 3c), indicating the mitochondrial dysfunction by OXPHOS inhibition. Interestingly, Lip(MA+Met)‐R1 showed a slightly decreased OCR curve compared with the Lip(Met)‐R1 group, indicating that MA‐mediated chemotherapy had a mild effect on mitochondrial dysfunction. In addition, both Lip(Met)‐R1 and Lip(MA+Met)‐R1 showed Met concentration‐dependent respiration inhibition toward MDA‐MB‐231 cells (Figure 3d and Figure S15). These results demonstrate that LNPs could destroy the mitochondrial function of TNBC through metabolic regulation, which plays an important role in PD‐L1 and TGF‐β1 production. The mitochondrial damage was evaluated by monitoring the changes of mitochondrial membrane potential (MMP) using the JC‐1 probe, which emits red fluorescence of aggregates at high MMP and green fluorescence of monomers at low MMP. As shown in Figure 3g, obvious green fluorescence was presented after Lip(Met)‐R1 and Lip(MA+Met)‐R1 treatments, indicating reduced MMP and severe mitochondrial damage.

According to previous reports [34, 35], Met‐mediated OXPHOS inhibition can induce increased phosphorylation of AMP‐activated protein kinase (p‐AMPK). The expression levels of AMPK phosphorylation are negatively correlated with the expression of membrane and intracellular PD‐L1 and the secretion of TGF‐β1, thereby leading to enhanced T lymphocyte infiltration and amplified immune activation (Figure 3a). The related molecular mechanisms were investigated by Western blot assay after various treatments. Lip(Met)‐R1 and Lip(MA+Met)‐R1 treated MDA‐MB‐231 cells showed remarkably augmented p‐AMPK expression compared with the control group and Lip(MA)‐R1 treated group, accompanied by obvious downregulation of PD‐L1 and TGF‐β1 (Figure 3e, f). In addition, Lip(MA+Met)‐R1 treatment led to downregulation of hypoxia inducible factor‐1α expression (HIF‐1α), which is beneficial for alleviating T cell exhaustion and improving immune response in the immunosuppressive TME. Quantitative analysis by flow cytometry revealed a distinct downregulation of membrane‐localized PD‐L1 in Lip(MA+Met)‐R1 treated group, with an approximately 7.74‐fold decrease of PD‐L1‐positive cell proportion compared to the control group (Figure 3h). The expression of these T cell inhibitory factors was further visualized by immunofluorescence images. Compared with the control group and Lip(MA)‐R1 treated group, the Lip(MA+Met)‐R1 treated group showed remarkably attenuated green fluorescence of PD‐L1 and TGF‐β1 (Figure 3i,j). These results demonstrate that Lip(MA+Met)‐R1 can serve as a targeted immune adjuvant to sensitize immunotherapy through mitochondrial metabolism reprogramming.

### 2.5 In Vitro PMBC Mediated Cell Killing, Immune Activation, and Degranulation Effect Evaluation

**FIGURE 4:**
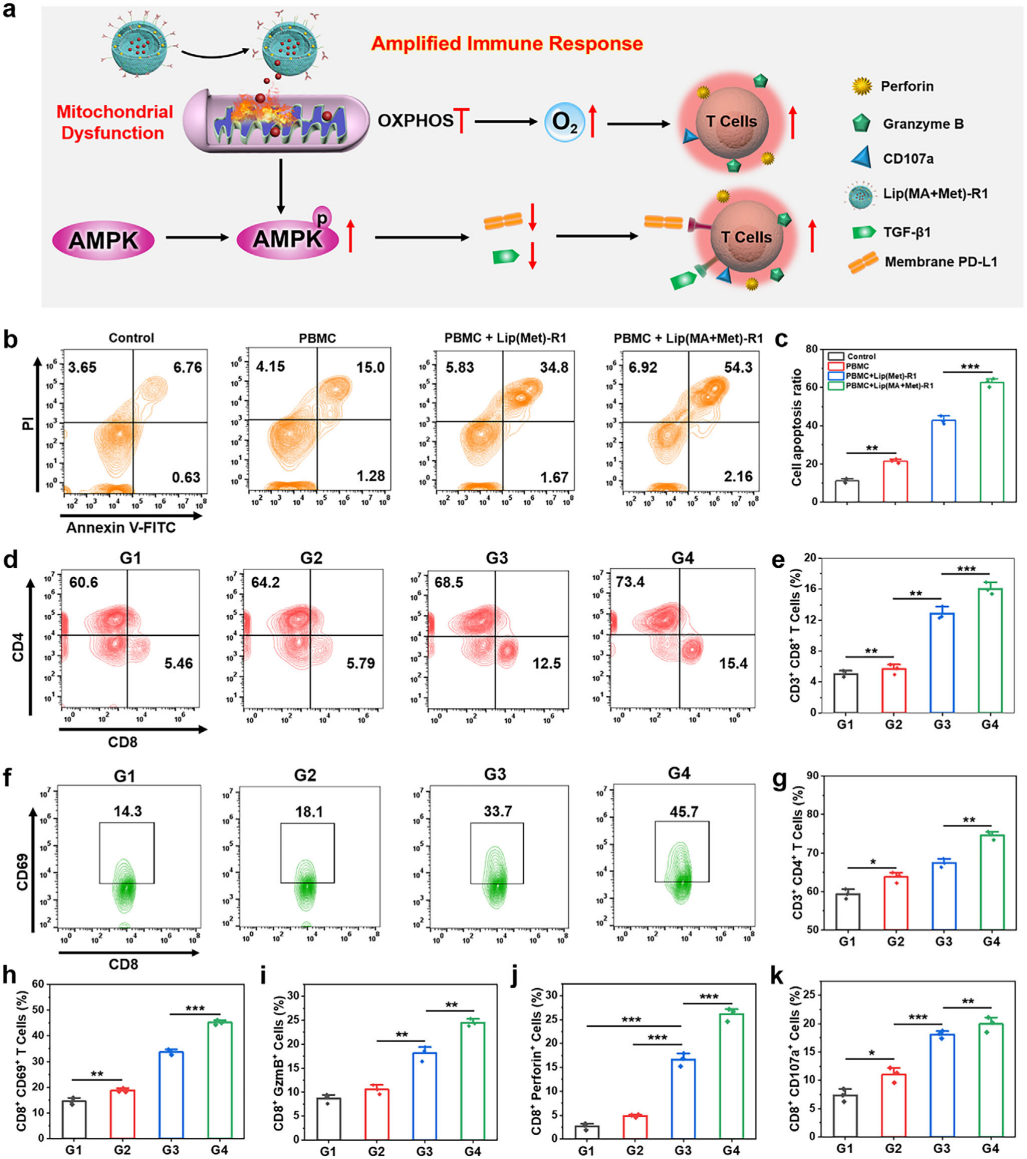
In vitro immune activation and anti‐tumor cell killing efficacy evaluation. (a) Schematic diagram of the amplified immune response mediated by Lip(MA+Met)‐R1. (b) Flow cytometry analysis and (c) statistical data of cell apoptosis of MDA‐MB‐231 cells undergoing different treatments. (d) Flow cytometry and (e) (g) quantification of CD8^+^ T cells and CD4^+^ T cells in different PBMC treatment groups. (f) Flow cytometry analysis and (h) statistical data of activated CD8^+^ T cells in different PBMC treatment groups. (i‐k) The secretion and expression of granzyme B, perforin, and CD107a in different treatment groups. G1: PBMC negative control group, G2: PBMC treatment, G3: PBMC+Lip(Met)‐R1, G4: PBMC+Lip(MA+Met)‐R1. n = 3 per group. ^*^ *p* < 0.05, ^**^ *p* < 0.01 and ^***^ *p* < 0.001.

Mitochondrial OXPHOS inhibition may provoke an amplified immune response through the inhibition of membrane‐located PD‐L1, along with TGF‐β1 suppression and hypoxia relief (Figure 4a). Human peripheral blood mononuclear cells (PBMCs) were used to evaluate Lip(MA+Met)‐R1 mediated immune activation and tumor cell killing effect against TNBC. MDA‐MB‐231 cells pretreated with Lip(Met)‐R1 and Lip(MA+Met)‐R1 were incubated with PBMC for 24 h. Apoptosis assay revealed that the PBMC+Lip(Met)‐R1 treated group presented a higher cell apoptosis rate compared with the group without any treatment and the group treated with PBMC alone (Figure 4b), which may be attributed to the boosted T cell activation by the downregulation of PD‐L1 and TGF‐β1, enabling the more efficient eradication of tumor cells. PBMC+Lip(MA+Met)‐R1 treatment exhibited the highest apoptotic rate, increasing by approximately 3.09 fold compared to that of the PBMC alone group (Figure 4c), attributed to the combination efficiency of chemo‐immunotherapy. T cell activation and degranulation effects were further evaluated by flow cytometry analysis. As shown in Figure 4d, PBMC+Lip(Met)‐R1 and PBMC+Lip(MA+Met)‐R1 treatments induced significantly more generation of CD3^+^ CD4^+^ and CD3^+^ CD8^+^ T cells. In particular, the population of CD3^+^ CD8^+^ T cells in PBMC+Lip(MA+Met)‐R1 group increased by about 2.66 fold compared with PBMC treatment alone (Figure S23, Figure 4e, g). Moreover, more activated CD8^+^ T cells were detected in the Lip(Met)‐R1 and Lip(MA+Met)‐R1 treated groups (Figure 4f, h). This may be due to the ability of Met to promote T cell activation by inhibiting the expression of PD‐L1 and TGF‐β1 of tumor cells, thereby enhancing T cell‐mediated immune response. The degranulation detection verified the highest secretion and expression of granzyme B, perforin, and CD107a in PBMC+Lip(MA+Met)‐R1 treated group (Figure S16, Figure 4i–k), thereby inducing the rapid apoptosis of tumor cells. These results confirm that LNPs can provoke amplified immune activation and tumor cell killing through immunosuppressive TME remodeling induced by mitochondrial metabolism regulation.

### 2.6 In Vitro and In Vivo Targeted NIR‐II Fluorescence Imaging

**FIGURE 5:**
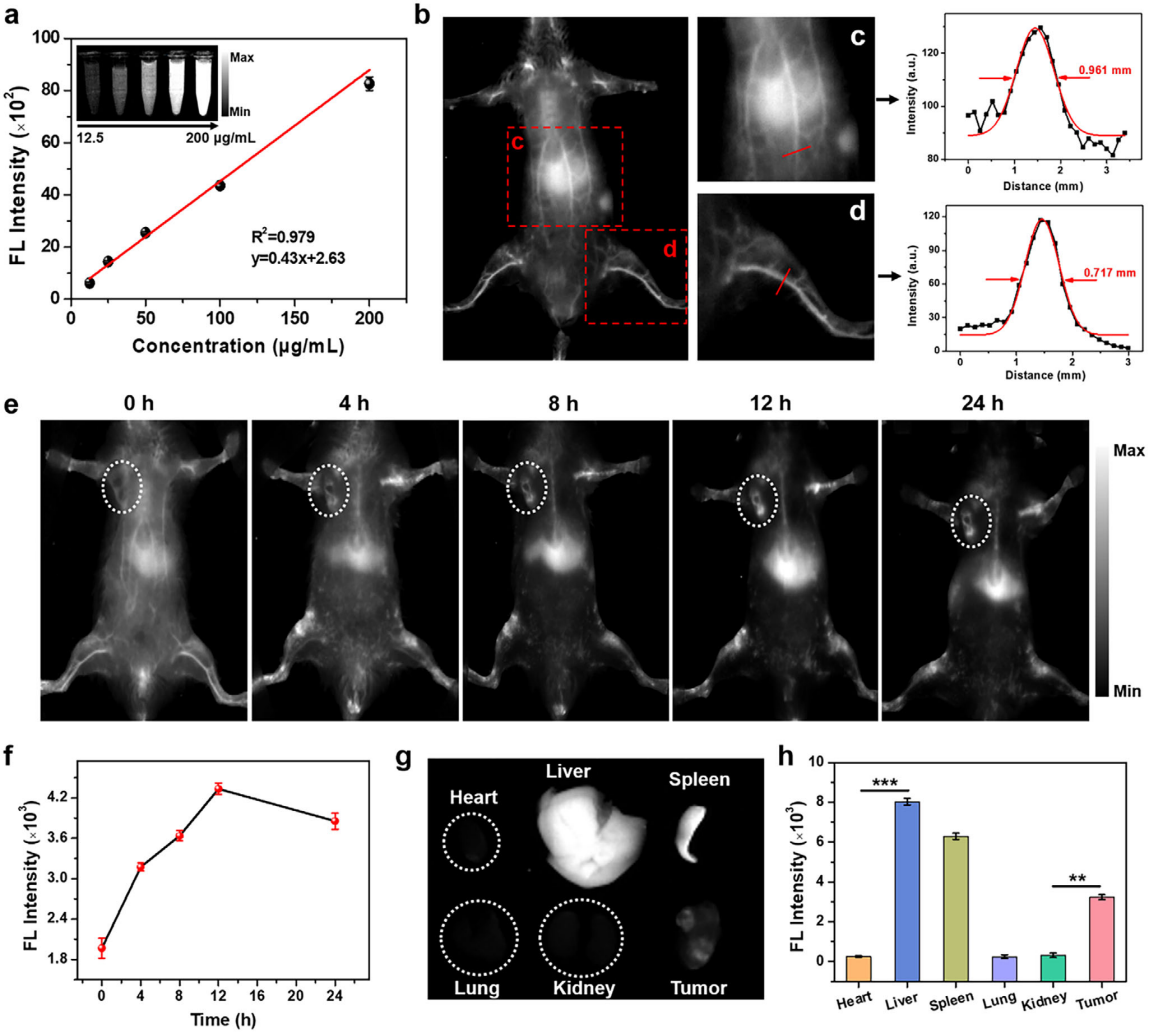
In vitro and in vivo NIR‐II fluorescence imaging. (a) NIR‐II fluorescence images of OTQ‐doped LNPs aqueous solutions. (b) NIR‐II fluorescence images of the systemic vasculature of mice after injection with LNPs via the tail vein. Fluorescence intensity analysis of labeled vessels in the (c) abdominal and (d) hindlimb vessels. (e) NIR‐II fluorescence images of MDA‐MB‐231 tumor‐bearing mice at different time points after intravenous injection with LNPs via the tail vein. (f) The quantitative detection of fluorescence intensity at tumor locations at various time points. (g) NIR‐II fluorescence images and (h) fluorescence intensity analysis of major organs and tumors at 24 h post‐injection. ^**^ *p* < 0.01 and ^***^ *p* < 0.001.

To achieve precise localization and diagnosis of deep‐seated tumors before the combination therapy, NIR‐II fluorescence imaging of tumors was performed. OTQ‐doped LNPs aqueous solution showed a concentration‐dependent enhancement of NIR‐II fluorescence signal (Figure 5a). The immediate NIR‐II fluorescence imaging of mice after injection via tail vein clearly delineated the systemic vascular distribution with high signal‐to‐background ratios (SBR) (Figure 5b). The imaging resolution of the marked abdominal blood vessel and hindlimb vessel reached 0.961 and 0.717 mm, respectively, through Gaussian fitting of the fluorescence intensity curves (Figure 5c, d). After tail vein injection of LNPs, NIR‐II fluorescence imaging of MDA‐MB‐231 tumor‐bearing mice indicated that the LNPs gradually accumulated from the blood vessels to the liver and tumor locations over time (Figure 5e). The NIR‐II fluorescence of the tumor site showed a time‐dependent enhancement with a peak at 12 h post‐injection (Figure 5f), implying the optimal enrichment time point of LNPs in the tumor. After 24 h post‐injection, NIR‐II fluorescence imaging of major organs and tumors suggested the preferential retention of LNPs in the liver, spleen, and tumor, which still retained higher fluorescence signals compared with other organs (Figure 5g, h). These results suggest that LNPs loaded with NIR‐II polymers can serve as a high‐resolution imaging agent for precise diagnosis of deep‐seated tumors, providing key information for subsequent combination therapy and efficient intervention.

### 2.7 In Vivo Anti‐Tumor Combination Therapy

**FIGURE 6:**
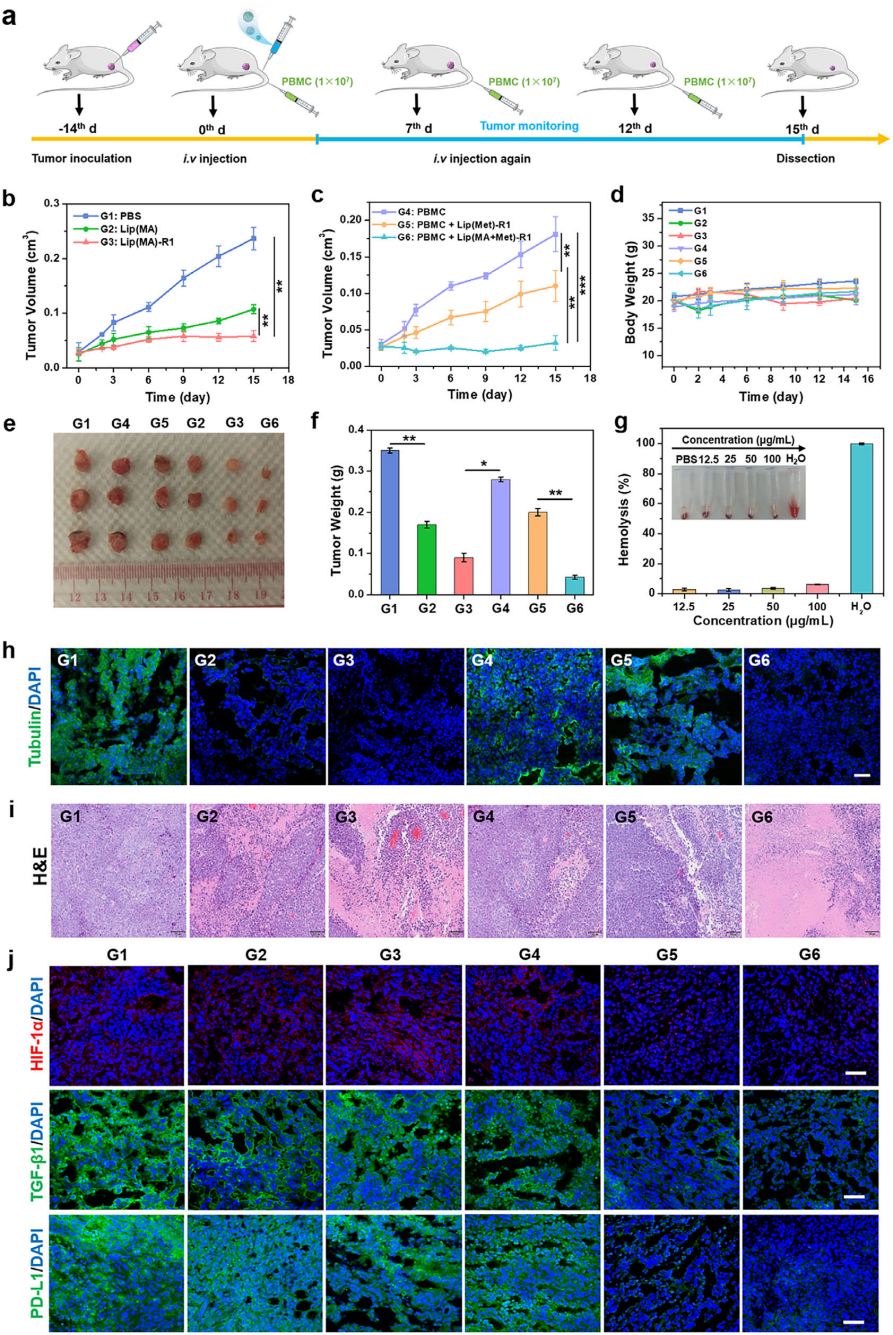
In vivo anti‐tumor combination therapy. (a) Schematic diagram of the TNBC tumor treatment process. (b) (c) Tumor growth curves of MDA‐MB‐231 tumor‐bearing mice after various treatments within 15 days (5 mice/group). (d) Body weight changes of MDA‐MB‐231 tumor‐bearing mice during 15 days (n = 5 per group). (e) Photographs and (f) tumor weight of excised MDA‐MB‐231 tumors after 15 days of treatment (n = 5 per group). (g) Hemolysis assay of Lip(MA+Met)‐R1 (n = 3 per group). (h) Immunofluorescence images of tubulin expression in tumor tissues after various treatments. Scale bar: 50 µm. (i) H&E staining images of tumor tissues. Scale bar: 100 µm. (j) Immunofluorescence images of HIF‐1α, TGF‐β1 and PD‐L1expression in tumor tissues after 15 days of treatment. Scale bar: 50 µm. G1: PBS, G2: Lip(MA), G3: Lip(MA)‐R1, G4: PBMC, G5: PBMC+Lip(Met)‐R1, G6: PBMC+Lip(MA+Met)‐R1. ^*^ *p* < 0.05, ^**^ *p* < 0.01 and ^***^ *p* < 0.001.

To evaluate the combination therapeutic effect of LNPs toward TNBC, MDA‐MB‐231 tumor‐bearing NSG mice were subjected to different treatments, followed by evaluation of immune activation effects by injection with PBMC via the tail vein (Figure 6a). The antibody‐mediated targeted chemotherapeutic effect was first evaluated. According to tumor growth curves, the lipid formation of MA exhibited significant tumor growth inhibition (Figure 6b), indicating tubulin inhibition mediated tumor killing efficiency. Moreover, ROR1 Ab conjugation further enhanced tumor eradication in Lip(MA)‐R1 treated group, attributed to the ROR1 Ab‐mediated targeted binding of LNPs toward tumor cells. This confirms the outstanding targeted chemotherapeutic effect of ADCs against TNBC. In addition, PBMC+Lip(Met)‐R1 injected group delayed tumor growth to some extent (Figure 6c), ascribed to the boosted immune activation by Met induced PD‐L1 and TGF‐β1 co‐depression. In particular, the highest tumor growth inhibition was monitored in the PBMC+ Lip(MA+Met)‐R1 treated group, with an inhibition rate of approximately 82.32% relative to the PBMC alone treated group, verified by the smallest tumor size and the lowest tumor weight after 15 days of treatment (Figure 6e, f). The immunofluorescence staining exhibited reduced tubulin expression in the Lip(MA), Lip(MA)‐R1, and PBMC+Lip(MA+Met)‐R1 treated groups (Figure 6h and Figure S19a), ultimately leading to tumor apoptosis. H&E staining revealed apparent tumor necrosis areas in the treatment groups, especially in the PBMC+Lip(MA+Met)‐R1 group (Figure 6i). These results verify that Lip(MA+Met)‐R1 can achieve a combination therapeutic effect through targeted chemotherapy and amplified immune response induced by tumor metabolism reprogramming.

The biocompatibility of Lip(MA+Met)‐R1 was further evaluated. The body weight of mice in all groups remained almost stable without major fluctuations during the 15‐day treatment period (Figure 6d). H&E staining images of major organs demonstrated negligible pathological abnormality or organ damage in the PBMC+Lip(MA+Met)‐R1 treated group (Figure S17). After 15 days of treatment, the serum samples of mice in all groups were collected for blood biochemical analysis. There were no significant differences in the expression of liver/kidney function indicators after various treatments (Figure S18), including alanine aminotransferase (ALT), aspartate transaminase (AST), creatinine (Crea), and urea, which was further confirmed by a low hemolysis rate (Figure 6g). These results confirm the biological security of LNPs‐mediated combination therapy.

### 2.8 In Vivo Immune Remodeling and Immune Activation

**FIGURE 7:**
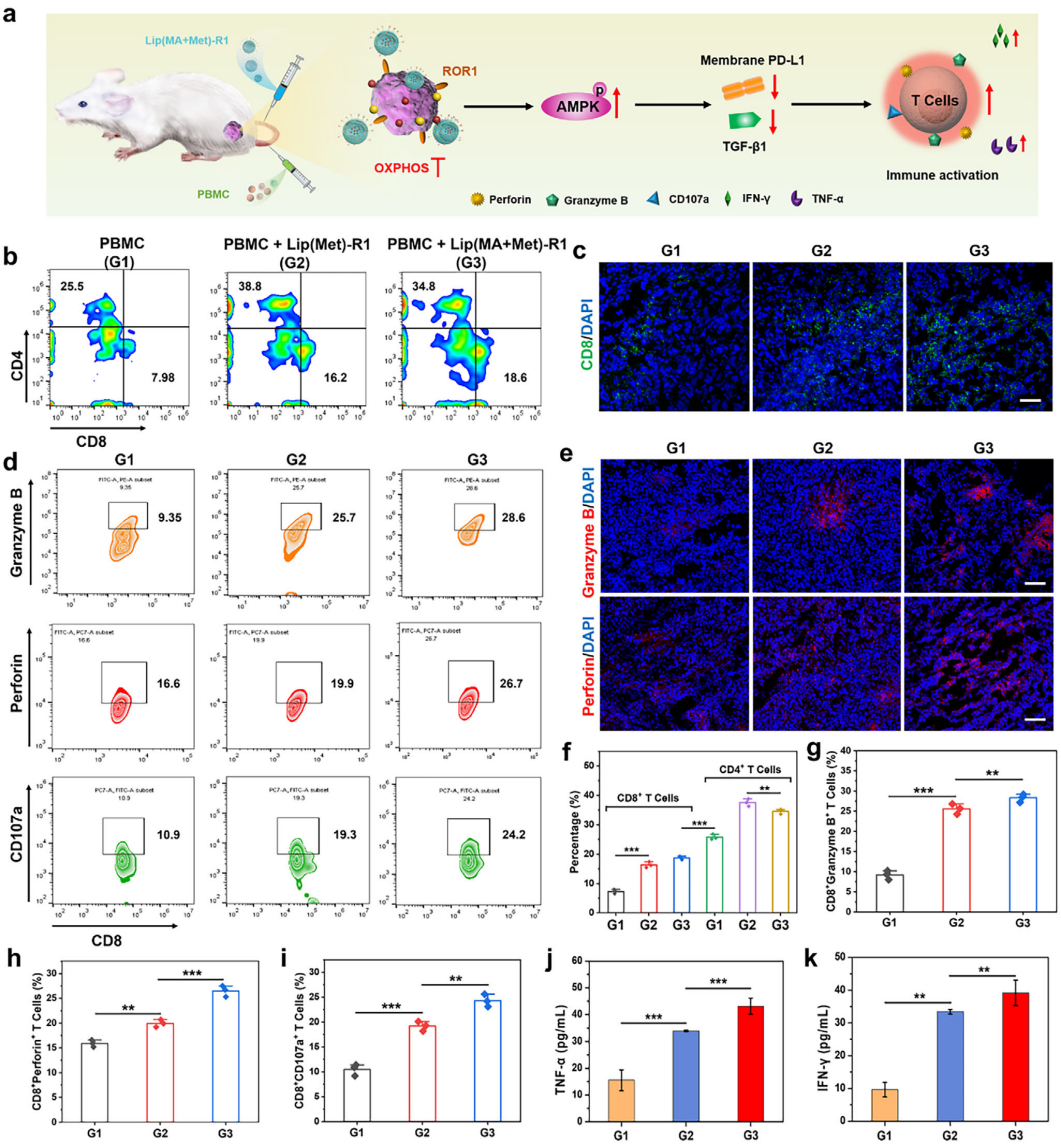
In vivo immune remodeling and immune activation. (a) Schematic diagram of Lip(MA+Met)‐R1 mediated amplified immune response. (b) Flow cytometric analysis and (f) statistical data of CD4^+^ T cells and CD8^+^ T cells within TNBC tumors from PBMC‐injected groups (n = 3 per group). (c) Immunofluorescence images of CD8^+^ T cells in tumor tissues. Scale bar: 50 µm. (d) Flow cytometric analysis and (g‐i) quantification of granzyme B, perforin, and CD107a within tumor tissues after various treatments for 15 days (n = 3 per group). (e) Immunofluorescence images of granzyme B and perforin within tumor tissues. Scale bar: 50 µm. The secretion of cytokine (j) TNF‐α and (k) IFN‐γ in the serum of mice after 15 days of treatment (n = 3 per group). G1: PBMC, G2: PBMC+Lip(Met)‐R1, G3: PBMC+Lip(MA+Met)‐R1. ^**^ *p* < 0.01 and ^***^ *p* < 0.001.

After 15 days of treatment, all groups of tumor tissues were collected for the expression evaluation of immunosuppression‐related markers. As shown in Figure 6j and Figure S19, PBMC+Lip(Met)‐R1 and PBMC+Lip(MA+Met)‐R1 treated groups exhibited remarkably reduced fluorescence of HIF‐1α, TGF‐β1, and PD‐L1 compared with the other treatment groups, implying Met mediated reversal of immunosuppressive TME through mitochondrial OXPHOS inhibition. These results suggest that TME remodeling induced by mitochondrial metabolism regulation may promote the infiltration and immune activation of cytotoxic T lymphocytes, thereby amplifying the efficiency of the anti‐tumor immunotherapy (Figure 7a).

T lymphocyte infiltration analysis within tumor tissues showed that the populations of CD8^+^ T cells were significantly increased in the PBMC+Lip(Met)‐R1 and PBMC+Lip(MA+Met)‐R1 treated groups (Figure 7b), enhanced by 2.03 and 2.33 fold, respectively, compared with the PBMC alone treated group (Figure 7f). More fluorescence representing CD8^+^ T cells further verified improved infiltration of cytotoxic T lymphocytes in the PBMC+Lip(Met)‐R1 and PBMC+Lip(MA+Met)‐R1 treated groups (Figure 7c), which may be attributed to the suppression of immunosuppressive factors mediated by metabolism reprogramming. The degranulation assay showed the most secretion and expression of granzyme B, perforin, and CD107a in the PBMC+Lip(MA+Met)‐R1 treated group (Figure 7d), ascribed to the boosted infiltration and immune responses of T lymphocytes (Figure 7g–i). Immunofluorescence staining further exhibited the highest expression of degranulation markers through amplified immune activation mediated by Lip(MA+Met)‐R1 (Figure 7e and Figure S20). In addition, the detection of cytokines in mouse serum revealed obviously higher levels of TNF‐α and IFN‐γ in PBMC+Lip(Met)‐R1 and PBMC+Lip(MA+Met)‐R1 treated groups (Figure 7j, k). These results demonstrate the great potential of ROR1 Ab‐engineered LNPs for reshaping the immunosuppressive TME through tumor metabolism reprogramming, thereby potentiating chemo‐immunotherapy.

### 2.9 The Potential Mechanism of Lip(MA+Met)‐R1 Mediated Combination Therapy

**FIGURE 8:**
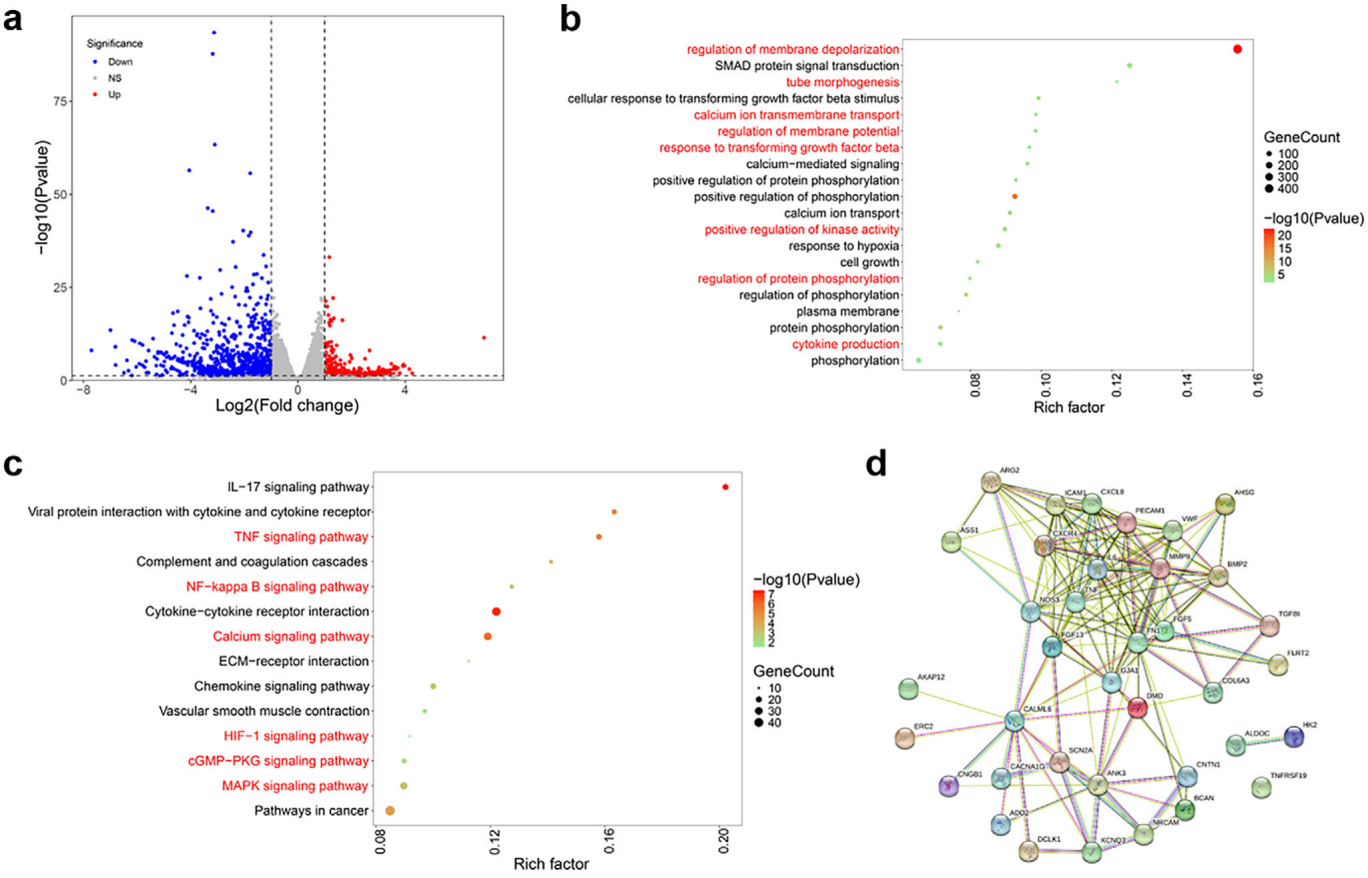
mRNA sequencing analysis. (a) Volcano map of DEGs between the PBMC group and PBMC+Lip(MA+Met)‐R1 group. (b) GO enrichment analysis and (c) KEGG pathway enrichment analysis of KEGs. (d) PPI based on DEGs in biological processes.

mRNA sequencing analysis was performed to investigate the potential therapeutic mechanism of Lip(MA+Met)‐R1 potentiating chemo‐immunotherapy. The volcano plots and heat maps shown in Figure 8a and Figure S21 revealed approximately 1525 differentially expressed genes (DEGs) between the PBMC group and the PBMC+Lip(MA+Met)‐R1 group, including 411 up‐regulated DEGs and 1114 down‐regulated DEGs. Gene Ontology (GO) enrichment analysis showed that PBMC+Lip(MA+Met)‐R1 treament altered genes related to tube morphogenesis, plasma membrane, cell growth and calcium ion transport in biological processes (Figure 8b), which may be ascribed to the MA‐mediated chemotherapeutic effect through tubulin inhibition, affecting the tube morphogenesis and transmembrane transport balance of calcium ions, ultimately leading to tumor cell apoptosis. In particular, mitochondria‐related genes were significantly altered, including membrane potential regulation, protein phosphorylation, and hypoxia response. These gene changes indicated that Met in Lip(MA+Met)‐R1 could alleviate the tumor hypoxic microenvironment and improve the expression of AMPK phosphorylation through mitochondrial OXPHOS inhibition. Improved AMPK phosphorylation further downregulated the expression of PD‐L1 and TGF‐β1, evidenced by the changes in TGF‐β‐related genes. Kyoto Encyclopedia of Genes and Genomes (KEGG) enrichment analysis verified PBMC+Lip(MA+Met)‐R1 mediated combination therapeutic effect through the changes of apoptosis‐related signaling pathways, such as the calcium signaling pathway, the cGMP‐PKG signaling pathway, and the MAPK signaling pathway (Figure 8c). In addition, Met‐mediated immune‐related signaling pathways were also activated, including the TNF signaling pathway, cytokine‐cytokine receptor interaction, and NF‐kappa B signaling pathway, achieving amplified immune output. The PBMC+Lip(MA+Met)‐R1 group exhibited more CD8^+^ T cell retention and infiltration, accompanied by lower production of regulatory T cells (Tregs) compared with the PBMC group (Figure S22), indicating an amplified immune response. The protein‐protein interaction (PPI) network revealed the key proteins involved in calcium binding and immune regulation, such as CALML6, IL6, TNF, and TGF‐β1 (Figure 8d). These results confirm that Lip(MA+Met)‐R1 could provoke amplified chemo‐immune output through TME remodeling mediated by mitochondrial metabolism reprogramming.

## 3 Conclusion

In summary, a novel LNPs with targeted TME reprogramming ability was prepared by thin film hydration method, utilizing liposomes to encapsulate clinical chemotherapeutic drugs and immunoadjuvants, followed by conjugation with human ROR1 antibody, for amplified chemo‐immunotherapy toward TNBC. During ROR1 antibody‐mediated active targeting and internalization into MDA‐MB‐231 cells, the LNPs enable visualization of blood vessels and tumors by NIR‐II fluorescence imaging, and the acidic TME triggers the dissociation of lipid nanomedicines from the antibody conjugate, inducing tumor cell apoptosis through cytotoxin MA‐mediated tubulin inhibition. More importantly, Met in liposomal formation reverses the immunosuppressive TME by downregulating the expression of PD‐L1 and TGF‐β1 induced by mitochondrial OXPHOS inhibition, thereby promoting the infiltration and immune activation of cytotoxic T lymphocytes and potentiating the combination efficiency of chemo‐immunotherapy. This work integrates NIR‐II fluorescence imaging‐guided chemotherapy and amplified immune activation into a single lipid nanoplatform modified by a human targeting antibody, providing a new strategy to enhance the combination anti‐tumor therapeutic efficacy against TNBC through tumor metabolism reprogramming.

## Conflicts of Interest

The authors declare no conflict of interest.

## Supporting information




**Supporting File**: advs74097‐sup‐0001‐SuppMat.docx

## Data Availability

The data that support the findings of this study are available from the corresponding author upon reasonable request.
