# Supplementary material for: Universal Antibody‐Engineered Lipid Nanoparticles Potentiate Chemo‐Immunotherapy Against Triple‐Negative Breast Cancer by Reprogramming Tumor Cell Metabolism
Source: Adv Sci (Weinh). 2026 Jan 27;13(19):e18468. doi: 10.1002/advs.202518468 (PMC13045483; doi:10.1002/advs.202518468)
Supplement: Supplementary file 1 — Supporting File: advs74097‐sup‐0001‐SuppMat.docx [file ADVS-13-e18468-s001.docx]

**Supporting information**

**Universal antibody-engineered lipid nanoparticles potentiate chemo-immunotherapy against triple-negative breast cancer by reprogramming tumor cell metabolism**

*Yeneng Dai, Jiaqi Wang, Yu Liu, Guanda Jiao, Yuheng Gu, Yang Liu, Shengyu Fu, Xing Fan, Jialin Li, Ziang Guo,* [*Kam Tong Leung*](https://www.nature.com/articles/s41375-019-0593-7#auth-Kam_Tong-Leung-Aff1)*, Lipeng Zhu,* Qi Zhao**

Cancer Centre, Institute of Translational Medicine, Faculty of Health Sciences, University of Macau, Macau SAR 999078, China.

E-mail: qizhao@um.edu.mo

MoE Frontiers Science Center for Precision Oncology, University of Macau, Taipa, Macau SAR 999078, China.

School of Life Sciences, Central South University, Changsha 410013, China.

E-mail: [zhuleaper@csu.edu.cn](mailto:zhuleaper@csu.edu.cn)

Department of Paediatrics, the Chinese University of Hong Kong, Hong Kong, China.

**Experimental section**

**Materials**

1,2-dipalmitoyl-sn-glycero-3-phosphocholine (DPPC), 1,2-distearoyl-sn-glycero-3-phosphoethanolamine-N-[amino(polyethylene glycol)-5000] (DSPE-PEG_5000_-NH_2_) and cholesterol were purchased from Shanghai Ponsure Biotech, Inc. Monomethyl auristatin E (MA) and metformin (Met) were bought from Bide Pharmatech Ltd. (Shanghai, China). (4,8-bis((2-ethylhexyl)oxy)benzo[1,2-b:4,5-b’]dithiophene-2,6-diyl)bis(trimethylstannane) (OT) and 6,7-Bis(4-(hexyloxy)phenyl)-4,9-di(thiophen-2-yl)- [1,2,5]thiadiazolo [3,4-g]quinoxaline (TTQ) were purchased from SunaTech Inc. Cell Counting Kit (CCK-8), Calcein AM/PI, Annexin V-FITC/PI apoptosis detection kit, JC-1 probe and MitoTracker Red CMXRos were obtained from Yeasen Biotechnology CO., Ltd. (Shanghai, China). APC-CD3, PE-CD4, PE-CD69, FITC-CD8, PE-Granzyme B, PE/Cy7-Perforin, PE-CD107a and FITC-CD107a were bought from BioLegend.

**Characterization**

^1^H NMR spectra of DSPE-PEG_5000_-CHO and NIR-II polymer OTQ was determined using the Bruker Ultra Shield Plus NMR instrument (400 MHz). HT7700 transmission electron microscope (100 KV) was used for morphology observation of nanoparticles and Nano-ZS ZEN3600 (Malvern) was used for detection of size distribution and zeta potential. The UV-visible absorption spectrum and NIR-II fluorescence emission spectrum were detected using Shimadzu UV-3600 spectrophotometer and NIR-II spectrophotometer (Fluorolog 3, Horiba), respectively. The fluorescence imaging of cells was detected utilizing a confocal laser scanning microscope (Carl Zeiss LSM710 Confocal with BiG module). AniView Phoenix Full Spectrum Animal *In Vivo* Imaging System (Guangzhou Biolight Biotechnology Co., Ltd.) was used for NIR-II fluorescence imaging *in vitro* and *in vivo*.

**Synthesis of NIR-II polymer OTQ**

NIR-II semiconducting polymer were synthesized via Still polymerization reaction. Briefly, (4,8-bis((2-ethylhexyl)oxy)benzo[1,2-b:4,5-b’]dithiophene-2,6-diyl)bis(trimethylstannane) (OT) (0.011 mmol, 8.50 mg) and 6,7-Bis(4-(hexyloxy)phenyl)-4,9-di(thiophen-2-yl)- [1,2,5]thiadiazolo [3,4-g]quinoxaline (TTQ) (0.011 mmol, 9.49 mg), bis(triphenylphosphine)palladium(II) dichloride (8 mg, 0.011 mmol) and 2,6-di-tertbutylphenol (5 mg, 0.024 mmol) were dissolved in toluene in a round-bottom flask, followed by recharged and degassed three times using nitrogen. The mixed solution was heated by an oil bath at 100 °C. After reaction for 1 h under continuous stirring, the mixture solution was added dropwise to methanol and the precipitate was collected by centrifugation. The precipitate was washed with methanol and dried under vacuum to obtain the polymer OT-TTQ (OTQ).

**Synthesis of DSPE-PEG_5000_-CHO**

15 mg 4-Formylbenzoic acid was dissolved in 3 mL DMF, followed by the addition of 1-(3-Dimethylaminopropyl)-3-ethylcarbodiimide hydrochloride (EDC, 10 mg) and N-hydroxysuccinimide (NHS, 10 mg) for reaction for 1 h under stirring. Then, 50 mg DSPE-PEG_5000_-NH_2_ was added to the mixed solution. After reaction for 24 h, the solution was dialyzed (MW: 3 KDa) to remove free 4-Formylbenzoic acid and then freeze-dried into powder for further use.

**Preparation of Lip(MA+Met)**

10 mg DSPE-PEG-CHO, 10 mg DPPC, 3 mg cholesterol and 10 μg monomethyl auristatin E (MA) were dissolved in 8 mL chloroform in a round bottom flask, followed by evaporated on a rotary evaporator at 40 ℃ for 30 min to form a lipid film. After added with 5 mL metformin (Met, 200 μg/mL) aqueous solution, the film was hydrated under continuous ultrasound, then the solution was ultrafiltered three times (10 KDa, 3000 rpm) to obtain LNPs Lip(MA+Met).

**Preparation of Lip(MA+Met)-R1**

To obtain human ROR1 antibody-coupled LNPs, 100 μg ROR1 antibody (ROR1 Ab) was added to the Lip(MA+Met) aqueous solution, followed by reaction for 24 h in a shaker (450 rpm). Then, the solution was dialyzed (300 KDa) overnight to obtain ROR1 Ab conjugated liposome Lip(MA+Met)-R1. To obtain rhodamine B (RhB) doped LNPs, 50 μL RhB aqueous solution (1 mg/mL) was added into the lipid film solution after rotary evaporation. In order to achieve NIR-II fluorescence imaging-guided tumor therapy, 300 μL NIR-II polymer OTQ solution (1 mg/mL, in tetrahydrofuran) was added into chloroform solution, and the polymer-doped Lip(MA+Met)-R1 was prepared by a similar procedur**e.**

**Fluorescence localization analysis and ROR1 expression evaluation**

The prepared RhB doped Lip(MA+Met)-R1 was incubated with Alexa-647 Goat anti-Human IgG antibody for 30 min at 4 ℃, followed by observed under a confocal laser scanning microscope (CLSM). In addition, MDA-MB-231 cells were incubated with free ROR1 Ab and RhB-doped Lip(MA+Met)-R1 for 12 h, respectively. After washed with PBS for three times, the cells were fixed with 4% paraformaldehyde for 30 min, then sequentially permeabilized with 0.25% Triton X-100 for 25 min and blocked with 2.5% BSA for 2 h. The cells were incubated with Alexa-647 Goat anti-Human IgG for 1 h. After staining the nucleus with Hoechst 33342, the cells were observed under CLSM.

To evaluate ROR1 antigen expression on MDA-MB-231 cells, ROR1 antibody (1 μg) was incubated with lung cancer cell line A549 cells, human colon cancer cell line COLO 205 cells, human colorectal adenocarcinoma cell line LS174T cells, human acute lymphoblastic leukemia cell line RS4:11 cells, human breast cancer cell line MCF-7 cells and triple-negative breast cancer cell line MDA-MB-231 cells for 1 h at 4 °C, respectively, followed by staining with Alexa-647 Goat anti-Human IgG. ROR1 expression on the surface of all cells was detected by flow cytometry.

**Acid-responsive ROR1 Ab release from Lip(MA+Met)-R1**

To investigate the acid-triggered separation behavior of the antibody from the liposomes, the release of ROR1 Ab was detected. The Lip(MA+Met)-R1 aqueous solution was transferred into a dialysis bag (MW: 300 KDa) and immersed in PBS solutions with different pH values ​​at 37 ℃. The external solution was collected at different time points and the release of ROR1 Ab was examined by ELISA assay based on the antigen binding.

**Cellular uptake and 3D spheroid penetration analysis**

To investigate ROR1 Ab mediated positive targeting ability, MDA-MB-231 cells were incubated with Lip(MA+Met) and Lip(MA+Met)-R1 doped with RhB, respectively. At 24 h, cellular uptake was analyzed by flow cytometry and intracellular fluorescence of RhB was observed under CLSM.

Lysosome escape behaviors were evaluated via fluorescence co-localization analysis of Lip(MA+Met)-R1 and lysosomes. Briefly, MDA-MB-231 cells were cultured with Rhodamine B (RhB)-doped Lip(MA+Met)-R1 (RhB: 2 μg/mL) for different times. Cells were washed with PBS and stained with LysoTracker Red at 37°C for 30 min, followed by fixed with paraformaldehyde for 20 min. After washed three times with PBS, the cells were observed under CLSM. Fluorescent colocalization of lysosomes and liposomes was analyzed using Image J software to obtain Pearson’s correlation coefficient (PPC).

To evaluate the antibody-mediated cell penetration efficiency, approximately 3000 MDA-MB-231 cells were seeded in a sphere-ultra-low adsorption surface 96-well plate (Thermo Fisher Scientific, USA), followed by centrifugation at 300 ×*g* for 5 min. After 7 days of incubation, Lip(MA+Met) and Lip(MA+Met)-R1 doped with RhB (RhB: 2 μg/mL) were added to the formed 3D multicellular spheroids, respectively. After incubation for 24 h, the cell spheroids were observed under CLSM and the fluorescence intensity at different spheroid depths was quantified.

**Cytotoxicity and tubulin inhibition analysis**

MDA-MB-231 cells were seeded in 96-well plates for incubation for 24 h. Then the cells were incubated for 12 h with different concentrations of Lip(MA), Lip(MA)-R1, Lip(Met)-R1 and Lip(MA+Met)-R1. After washed with PBS, 10 μL of CCK-8 solution was added to each well for further incubation for 2 h, and cell viability was detected by the absorbance at 450 nm using a multi-function microplate reader. The cell killing effects were further visualized by Calcein AM/PI staining and quantified by flow cytometry using Annexin V-FITC/PI apoptosis detection kit.

To evaluate the tubulin inhibition efficacy mediated by chemotherapeutic drugs MA, MDA-MB-231 cells were subjected to different treatments for 12 h. After washed with PBS three times, the cells were sequentially fixed with 4% paraformaldehyde, permeabilized with 0.25% Triton X-100 and blocked with 2.5% BSA. After washed with PBS three times, the primary antibody β-Tubulin Antibody (#T0023, Affinity) was added to the cells for overnight incubation at 4°C, followed by incubated with the secondary antibody FITC-labeled Goat Anti-Mouse IgG (H&L) (ab6785, Abcam) for 2 h. After washed with PBS three times, the cells were stained with Hoechst 33342 and observed under CLSM.

**Oxygen consumption rate (OCR) detection**

To investigate the inhibitory effect of LNPs on mitochondrial respiration, the OCR within mitochondria was measured using the Agilent Seahorse XF Cell Mito Stress Test Kit. Briefly, MDA-MB-231 cells were seeded in XF 96-well plates. After incubation for 24 h, the cell culture medium was refreshed with fresh medium containing Lip(Met)-R1 and Lip(MA+Met)-R1 with various concentrations, and the cells were further incubated for 12 h. The OCR was detected using an Agilent Seahorse XF Analyzer (Agilent, USA) according to the protocol provided by the manufacturer.

**Mitochondrial membrane potential detection**

To evaluate the mitochondrial depolarization, MDA-MB-231 cells were seeded in confocal culture dishes and then incubated for 24 h. The cells were incubated with Lip(MA)-R1, Lip(Met)-R1 and Lip(MA+Met)-R1 (MA: 200 ng/mL, Met: 100 μg/mL), respectively. After 12 h of incubation, the cells were washed with PBS and added with new culture medium containing JC-1 probe for 15 min of staining. The changes of intracellular mitochondrial membrane potential were detected by CLSM.

**The expression of AMPK, p-AMPK, TGF-β1 and PD-L1 *in vitro***

To evaluate the influence of LNPs on the expression of AMPK, pAMPK, TGF-β1 and PD-L1. MDA-MB-231 cells were seeded in 6-well plates, followed by treated with Lip(MA)-R1, Lip(Met)-R1 and Lip(MA+Met)-R1, respectively. The cells were lysed with radioimmunoprecipitation assay (RIPA) lysis buffer and proteins were extracted by centrifugation at 12,000 ×*g* for 5 min. After BCA quantification, proteins were separated using 10% SDS-PAGE and then transferred to a polyvinylidene fluoride (PVDF) membrane. After blocked with 5% skim milk powder for 2 h, the membrane was washed with PBST three times. The membranes were incubated with the corresponding primary antibodies overnight at 4 °C: AMPKα Rabbit mAb (#5831, Cell Signaling Technology), p-AMPKα Rabbit mAb (#2535, Cell Signaling Technology), PD-L1 Mouse Monoclonal Antibody (#BF8035, Affinity), TGF-β1 antibody (TA506583, ORIGENE) and β-Actin Mouse mAb (AC004, ABclonal). After washed with PBST, the membranes were incubated with the corresponding secondary antibodies labeled with the horseradish peroxide (HRP), and the protein bands were detected using an enhanced chemiluminescence (ECL) system.

The intracellular expression of TGF-β1 and PD-L1 was further visualized by immunofluorescence staining. Briefly, after various treatments, MDA-MB-231 cells were sequentially fixed with 4% paraformaldehyde for 25 min, permeabilized with 0.25% Triton X-100 for 25 min and blocked with 2.5% BSA for 2 h. After washed three times with PBS, the cells were incubated with the corresponding primary antibodies of TGF-β1 and PD-L1 overnight at 4°C, followed by incubated with the corresponding fluorescent-labeled secondary antibodies. After nuclear staining, intracellular fluorescence was detected under CLSM. In addition, the expression level of intracellular PD-L1 was quantified by flow cytometry analysis.

**PBMC-mediated tumor cell killing and immune activation**

The peripheral blood mononuclear cells (PBMC) were isolated from venous blood samples using lymphoprep. To evaluate the killing effect of PBMC towards TNBC through LNPs-mediated immune activation, MDA-MB-231 cells were seeded in 6-well plates and incubated for 24 h. Tumor cells were pretreated with Lip(Met)-R1 and Lip(MA+Met)-R1 for 6 h, respectively, followed by added with PBMC at a ratio of 10:1 (PBMC: tumor cells) after washed with PBS. After further incubation for 24 h, tumor cells were collected for apoptosis analysis using Annexin V-FITC/PI apoptosis detection kit. The cell supernatants were collected and centrifuged for T cell activation detection and degranulation effect evaluation.

**Animal models**

NSG mice were provided by Animal Research Core of Faculty of Health Sciences, University of Macau. All animal experiments were approved by the Institutional Animal Care and Use Committee of Macau University, and performed in accordance with the approved protocol (UMARE-041-2020) by the University of Macau Animal Ethics Committee.

***In vitro* and *in vivo* NIR-II fluorescence imaging**

NIR-II fluorescence imaging of Lip(MA+Met)-R1 aqueous solution with different OTQ concentrations was detected under 808 nm laser excitation using an AniView Phoenix Full Spectrum Animal *In Vivo* Imaging System (Guangzhou Biolight Biotechnology Co., Ltd.). To investigate the *in vivo* NIR-II fluorescence imaging effect of LNPs, healthy NSG mice were intravenously injected with OTQ doped Lip(MA+Met)-R1 solution (1 mg/mL) through the tail vein, and NIR-II images of mouse blood vessels were acquired within 10 min post-injection. The fluorescence intensity of the labeled blood vessels was analyzed by Image J software. For *in vivo* tumor localization, MDA-MB-231 tumor-bearing mice were intravenously injected with OTQ doped Lip(MA+Met)-R1 solution (1 mg/mL) through the tail vein. NIR-II fluorescence images of mice were detected at different time points, and the fluorescence intensity of the tumor was quantitatively analyzed. At 24 h post-injection, the major organs and tumors of the mice were collected for fluorescence distribution evaluation.

***In vivo* anti-tumor combination therapy of Lip(MA+Met)-R1**

To evaluate the efficiency of chemo-immunotherapy mediated by LNPs, MDA-MB-231 subcutaneous tumor model was established by injecting MDA-MB-231 cells (1 × 10^6^ cells) into the right flank of NSG mice with immunodeficiency. MDA-MB-231 tumor-bearing mice were randomly divided into 6 groups (5 mice/group) and received the following different treatments: G1: PBS, G2: Lip(MA), G3: Lip(MA)-R1, G4: PBMC, G5: PBMC+Lip(Met)-R1, G6: PBMC+Lip(MA+Met)-R1. The injection dose of MA was 3 mg/kg, the injection dose of Met was 5 mg/kg and the injection dose of PBMC was 1 × 10^7^ cells/mouse. After 7 days and 12 days of treatment, G4, G5 and G6 groups were injected with PBMC again. The tumor volume and mouse body weight of all groups were monitored every 3 days. After 15 days of treatment, all groups of mice were euthanized and tumors were collected for photographing and weighing, and then used for hematoxylin-eosin (H&E) staining together with the major organs of mice (heart, liver, spleen, lung and kidney). Tumor apoptosis was further detected by the terminal deoxynucleotidyl transferase dUTP nick end labeling (TUNEL) staining.

**Western blot analysis and immunofluorescence staining of tumor tissues**

To evaluate the expression levels of HIF-1α, PD-L1 and TGF-β1 in tumor tissues after various treatments, all groups of tumor tissues were collected and lysed with lysis buffer after 15 days of treatment. After sufficient homogenization, the supernatant was collected by centrifugation for western blot analysis. The proteins were separated by 10% SDS-PAGE and then transferred to a PVDF membrane. The membrane was blocked with 5% skim milk powder for 2 h, followed by incubated with the corresponding primary antibodies overnight at 4 ℃. After washed with PBST, the membrane was incubated with HRP-conjugated secondary antibodies for 2 h at room temperature. The protein expression was detected using a chemiluminescence (ECL) system.

The expression of HIF-1α, PD-L1 and TGF-β1 was further visualized by immunofluorescence staining. The tumor tissues were cut into slices with 10 μm using Cryostat (Leica CM5030 Cryostat). The slices were sequentially fixed with cold acetone, permeabilized with 0.25% Triton X-100 and blocked with 2.5% BSA. Then, the sections were incubated with primary antibodies overnight at 4°C, followed by incubation with corresponding fluorescence labeled secondary antibodies for 2 h. The expression levels of the proteins in tumor tissues were detected by CLSM and the fluorescence intensity was quantified using Image J software.

***In vivo* immune remodeling and immune activation**

To investigate the effect of ROR1 Ab-conjugated LNPs mediated immune amplification, tumor tissues in the PBMC group, PBMC+Lip(Met)-R1 group and PBMC+Lip(MA+Met)-R1 group were collected for T cell infiltration and immune activation analysis after 15 days of treatment. Briefly, tumor tissues were mechanically ground to obtain single-cell suspensions and washed with PBS. The cells were stained with corresponding fluorescence-labeled antibodies of CD3, CD4 and CD8. For the degranulation evaluation of cytotoxic T lymphocyte, the cells were stained with fluorescence-labeled CD3, CD8, granzyme B, perforin and CD107a. In addition, tumor tissues were also cut into sections to evaluate T cell infiltration and degranulation effects by immunofluorescence staining. Mouse sera were collected for cytokine (TNF-α and IFN-γ) detection using commercial ELISA kits.

**Statistical analysis**

The experiments were repeated at least three times. All results were obtained as means and standard deviations calculated from the measurements by repeated experiments. Data were analyzed via ANOVA and statistical significance was provided. (**p* < 0.05, ***p* < 0.01 and ****p* < 0.001)

**
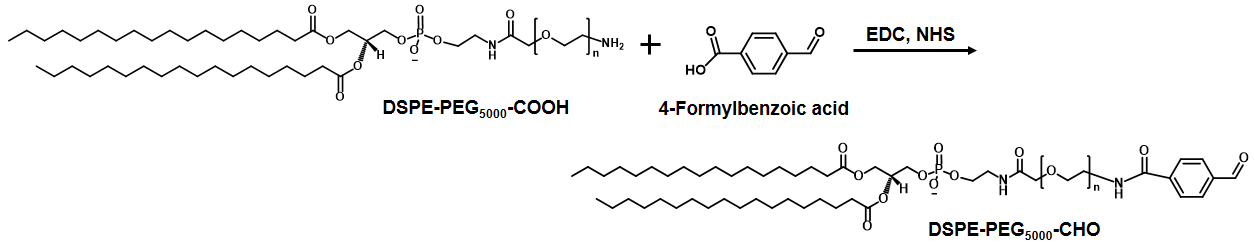
**

**Scheme. S1.** The Synthetic route of DSPE-PEG_5000_-CHO.

**
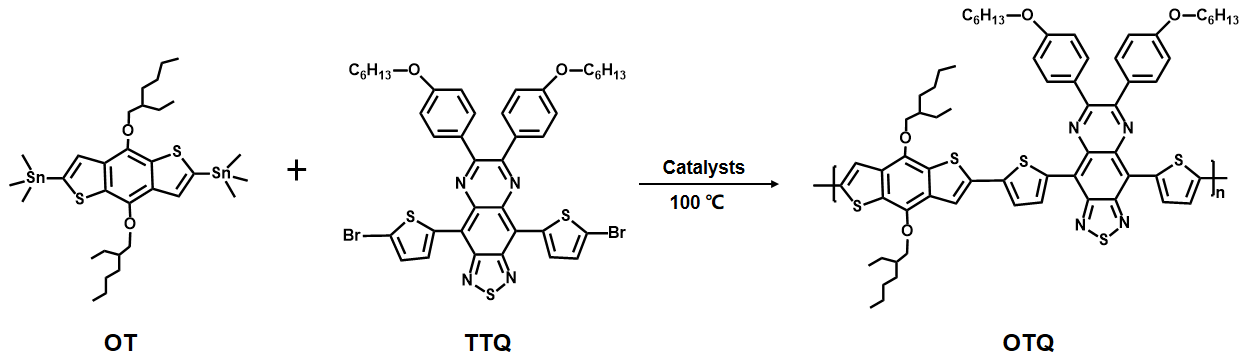
**

**Scheme. S2.** The synthetic route of the NIR-II semiconducting polymer OTQ. Catalysts: Bis(triphenylphosphine)palladium(II) dichloride and 2,6-Di-tertbutylphenol.

**
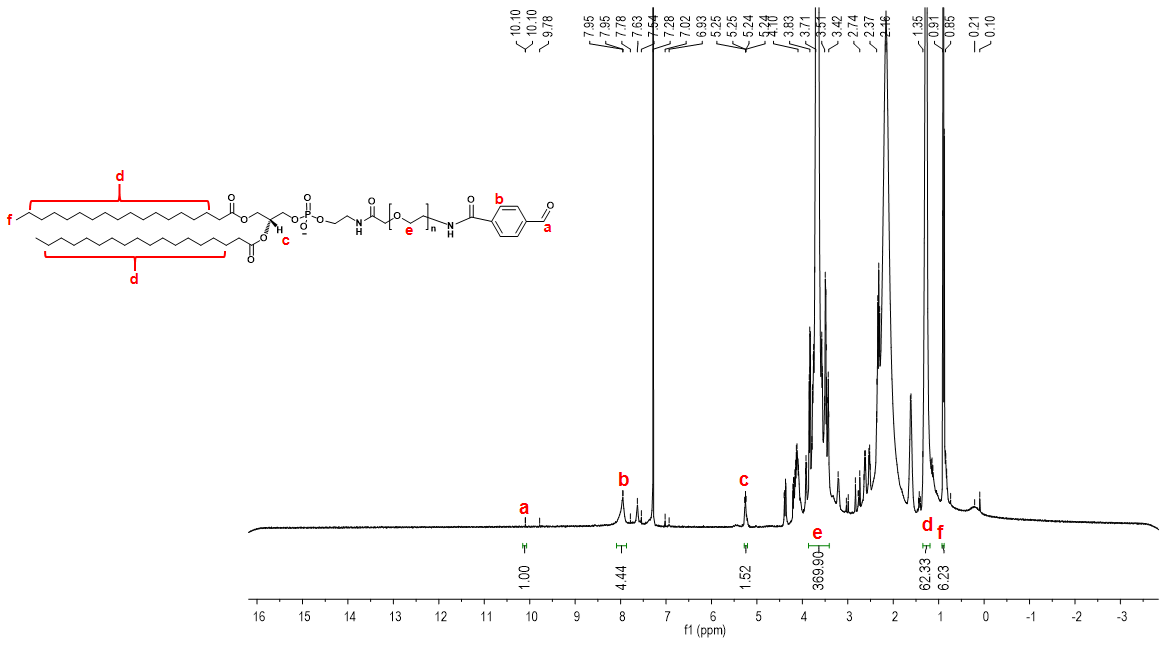
**

**Figure. S1.** ^1^H-NMR spectrum of DSPE-PEG_5000_-CHO.

**
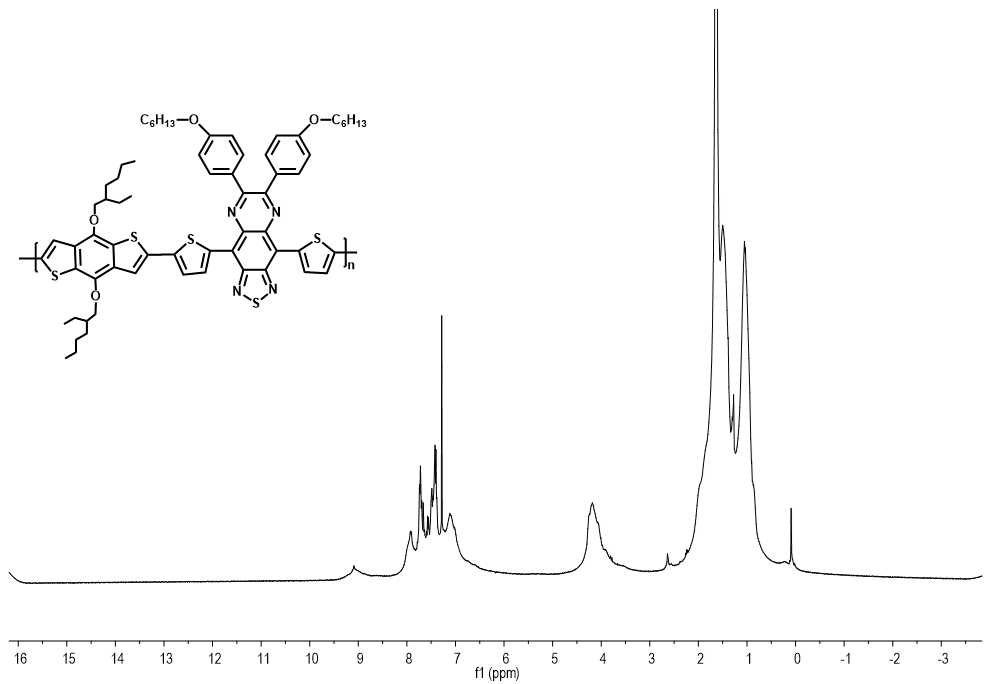
**

**Figure. S2.** ^1^H-NMR spectrum of NIR-II polymer OTQ.

**
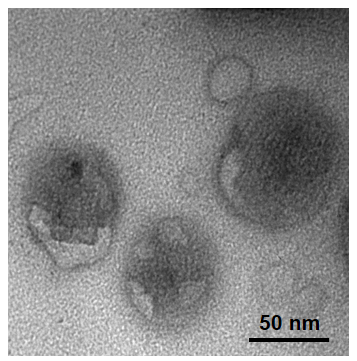
**

**Figure. S3.** TEM image of Lip(MA+Met).

**
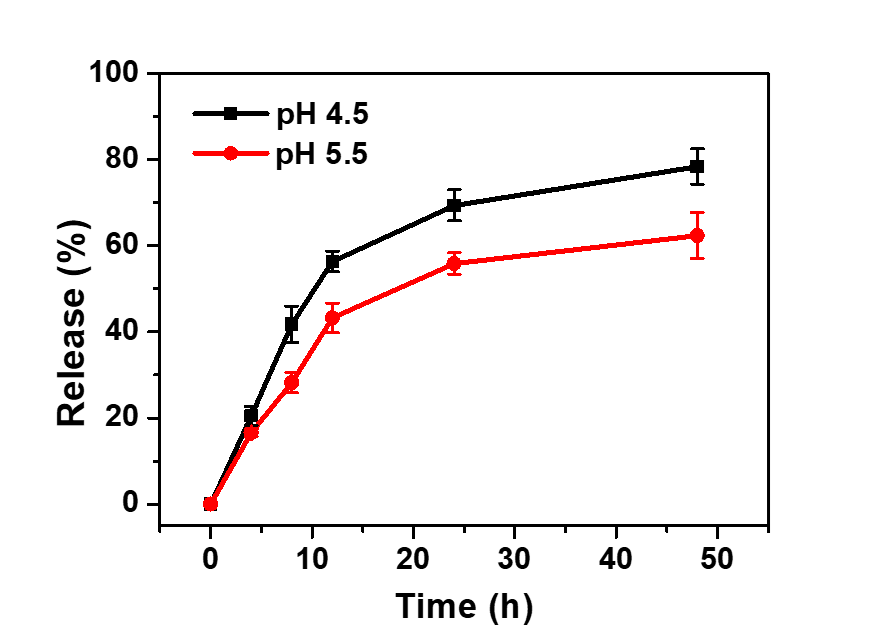
**

**Figure S4.** Cumulative release of ROR1 Ab from Lip(MA+Met)-R1 at different pH conditions.

**
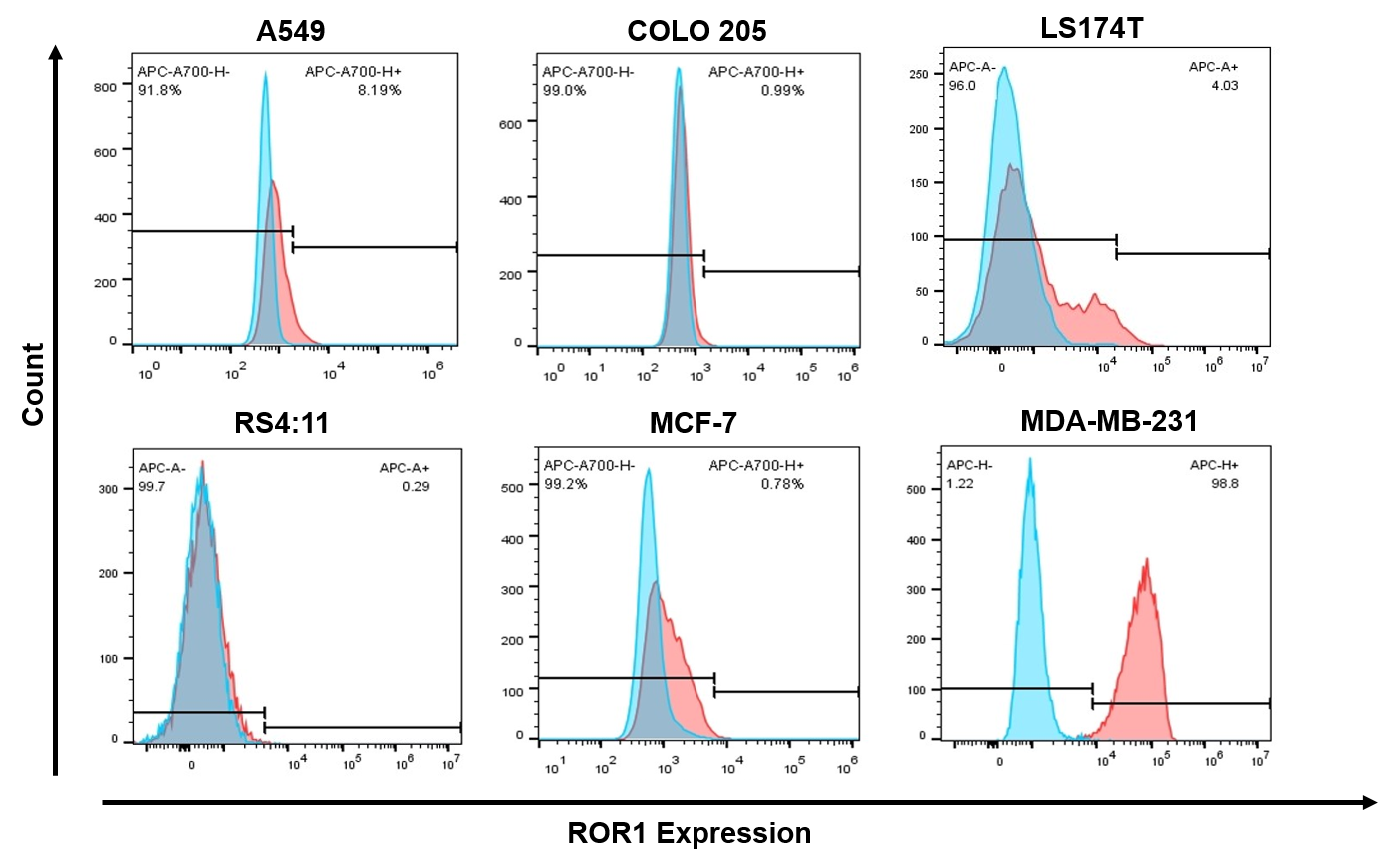
**

**Figure S5.** ROR1 expression in different cell lines by flow cytometry. lung cancer cell line A549 cells, human colon cancer cell line COLO 205 cells, human colorectal adenocarcinoma cell line LS174T cells, human acute lymphoblastic leukemia cell line RS4:11 cells, human breast cancer cell line MCF-7 cells and triple-negative breast cancer cell line MDA-MB-231 cells.

**
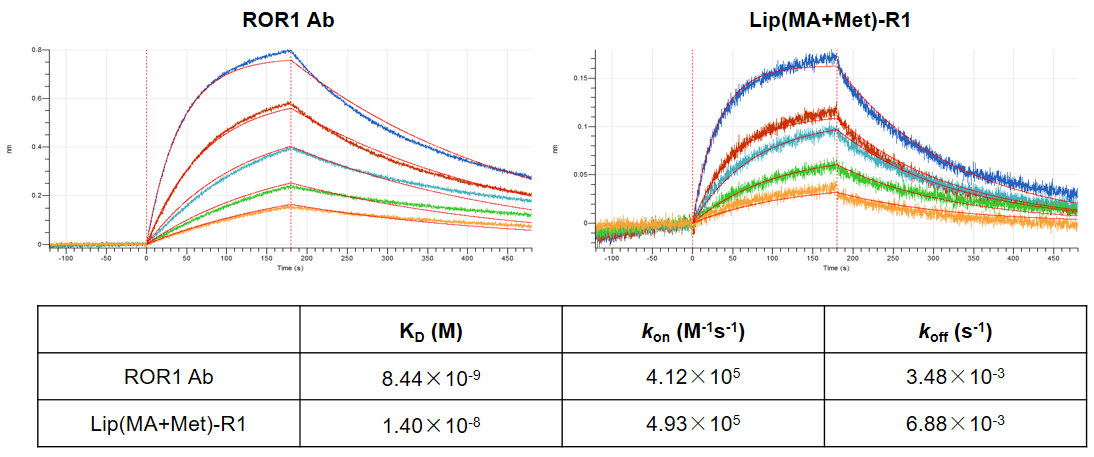
**

**Figure. S6.** The binding kinetics of ROR1 Ab and Lip(MA+Met)-R1 to ROR1 antigen by biolayer interferometry (BLI) assay. K_D_: equilibrium dissociation constant*, k*_on_: association rate constant, *k*_off_: dissociation rate constant.

**
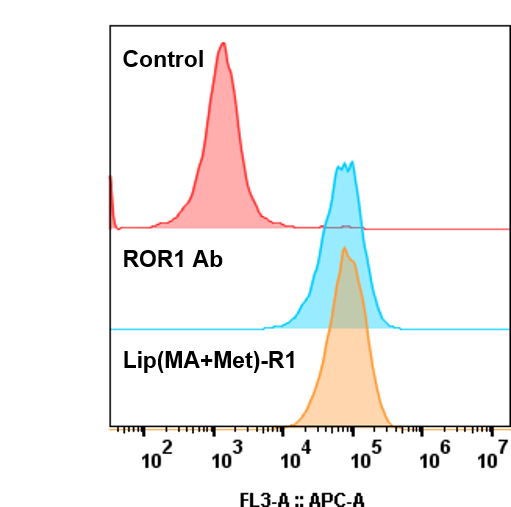
**

**Figure. S7.** Flow cytometric analysis of ROR1 Ab and Lip(MA+Met)-R1 binding to the surface of MDA-MB-231 cells after incubation for 1 h at 4 °C.

**
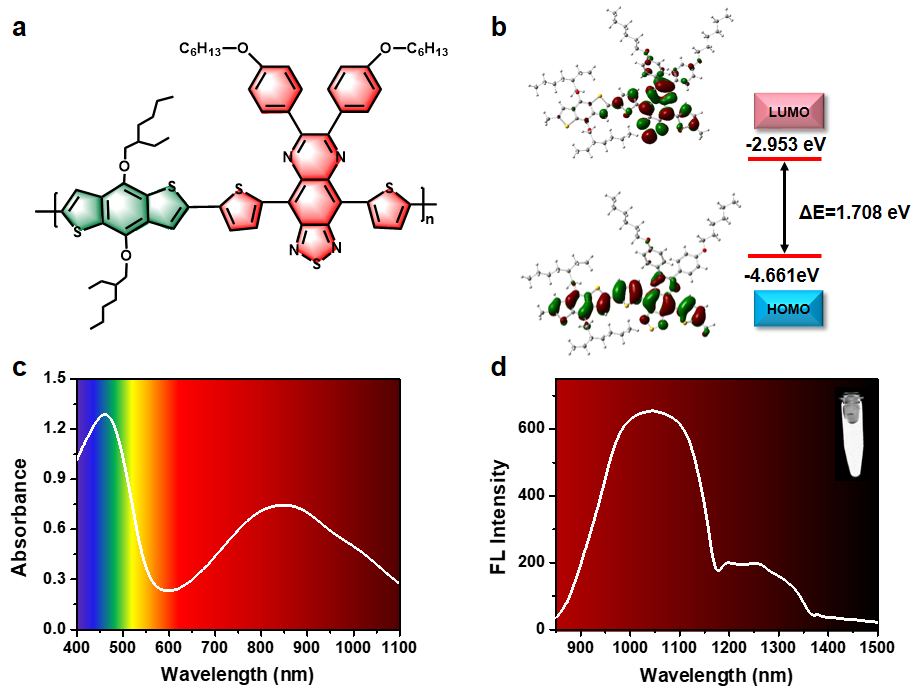
**

**Figure. S8.** (a) The chemical structure of the semiconducting polymer OTQ. (b) The highest occupied molecular orbitals (HOMO) and the lowest unoccupied molecular orbitals (LUMO) distribution of OTQ. (c) Absorption spectrum of OTQ and (d) fluorescence emission spectrum (insert: NIR-II fluorescence image) under 808 nm laser excitation.

**
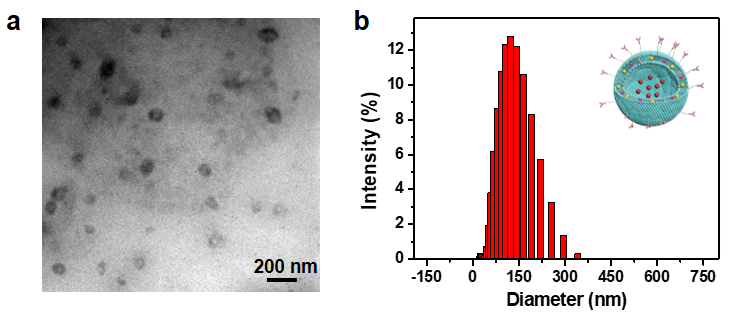
**

**Figure. S9.** (a) TEM image and (b) size distribution of OTQ-doped LNPs.


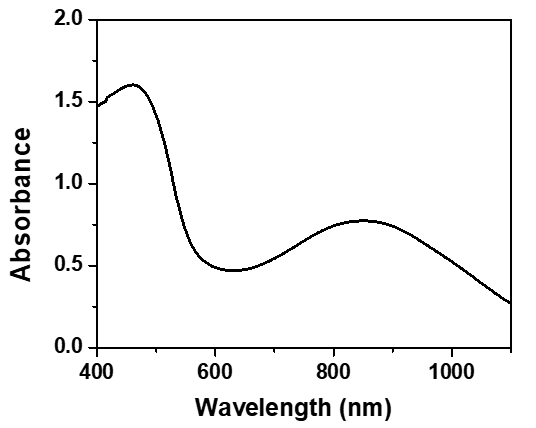


**Figure S10.** The absorption spectrum of OTQ-doped LNPs.

**
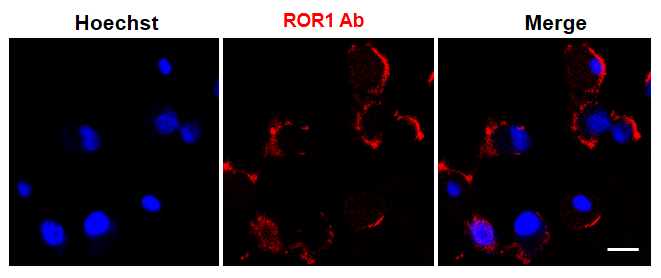
**

**Figure. S11.** The fluorescence localization of ROR1 Ab in MDA-MB-231 cells after incubation for 12 h. Scale bar: 20 μm.

**
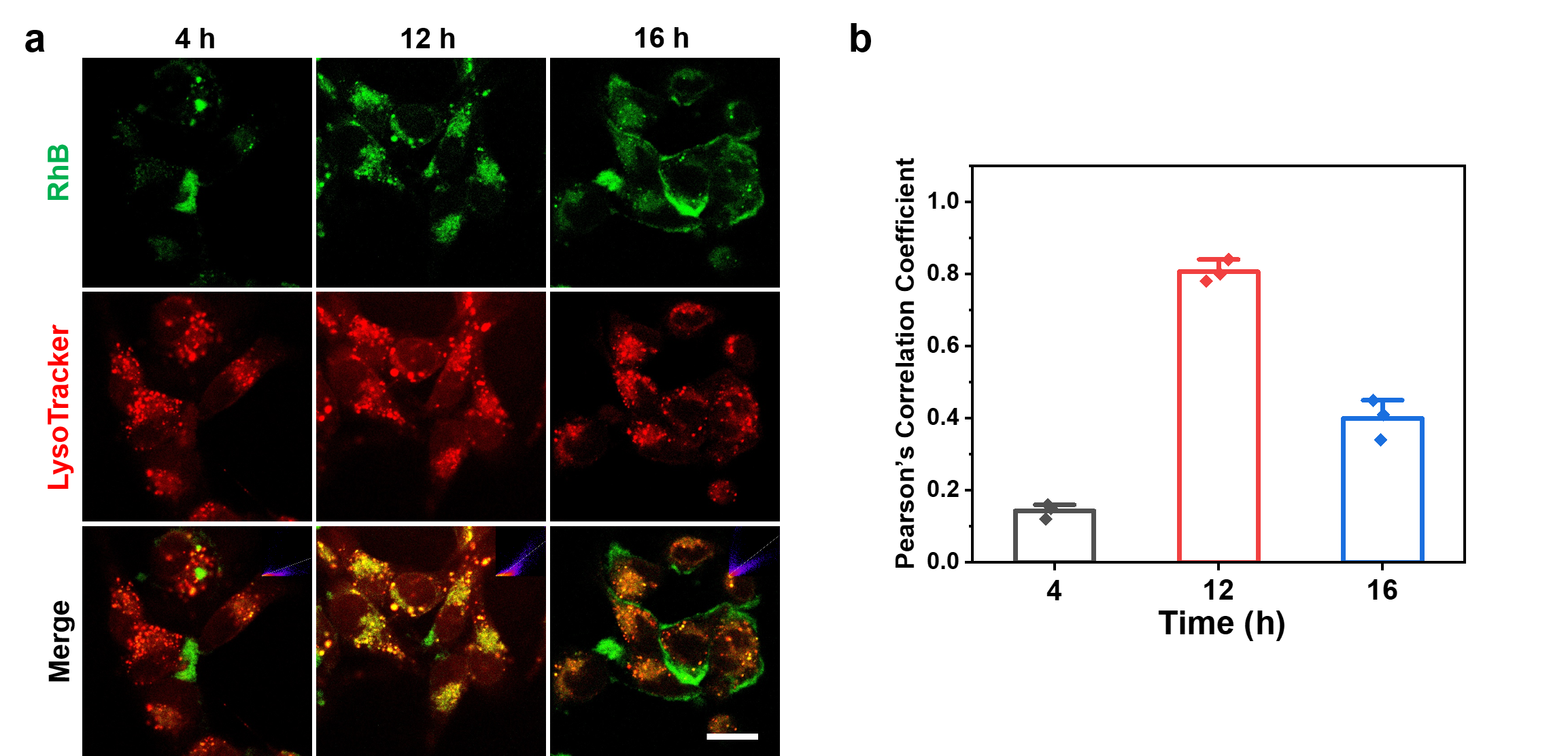
**

**Figure S12.** (a) The fluorescent images of RhB-labeled Lip(MA+Met)-R1 and lysosomes in MDA-MB-231 cells at different culture time points. Scale bar: 20 μm. (b) Pearson’s correlation coefficients based on the signals of RhB and lysotracker at various time points.

**
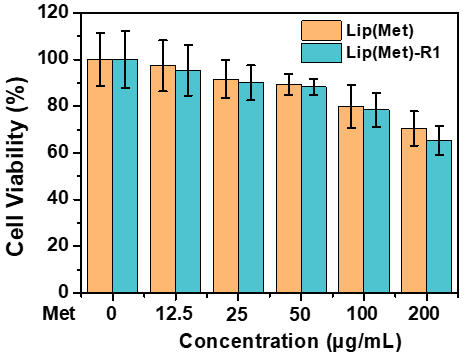
**

**Figure. S13.** Cell viability of MDA-MB-231 cells treated with Lip(Met) and Lip(Met)-R1 with various Met concentrations (n = 5 per group).

**
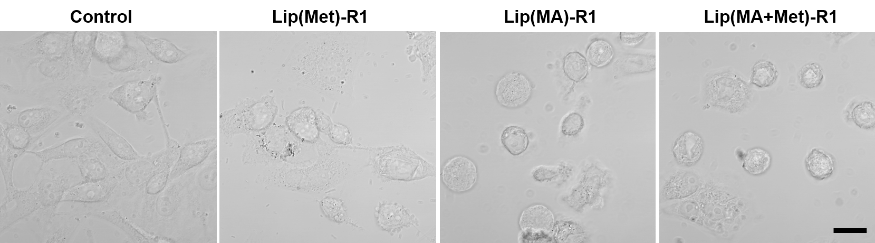
**

**Figure. S14.** Bright field images of MDA-MB-231 cells with different treatments. Scale bar: 20 μm.

**
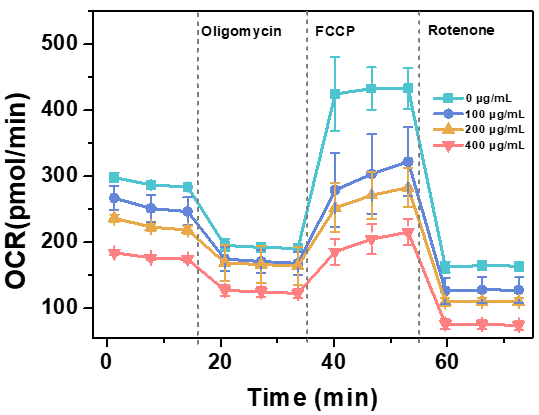
**

**Figure. S15.** Mitochondrial oxygen consumption rate (OCR) of MDA-MB-231 cells treated with Lip(Met)-R1 with various Met concentrations (n = 3 per group).

**
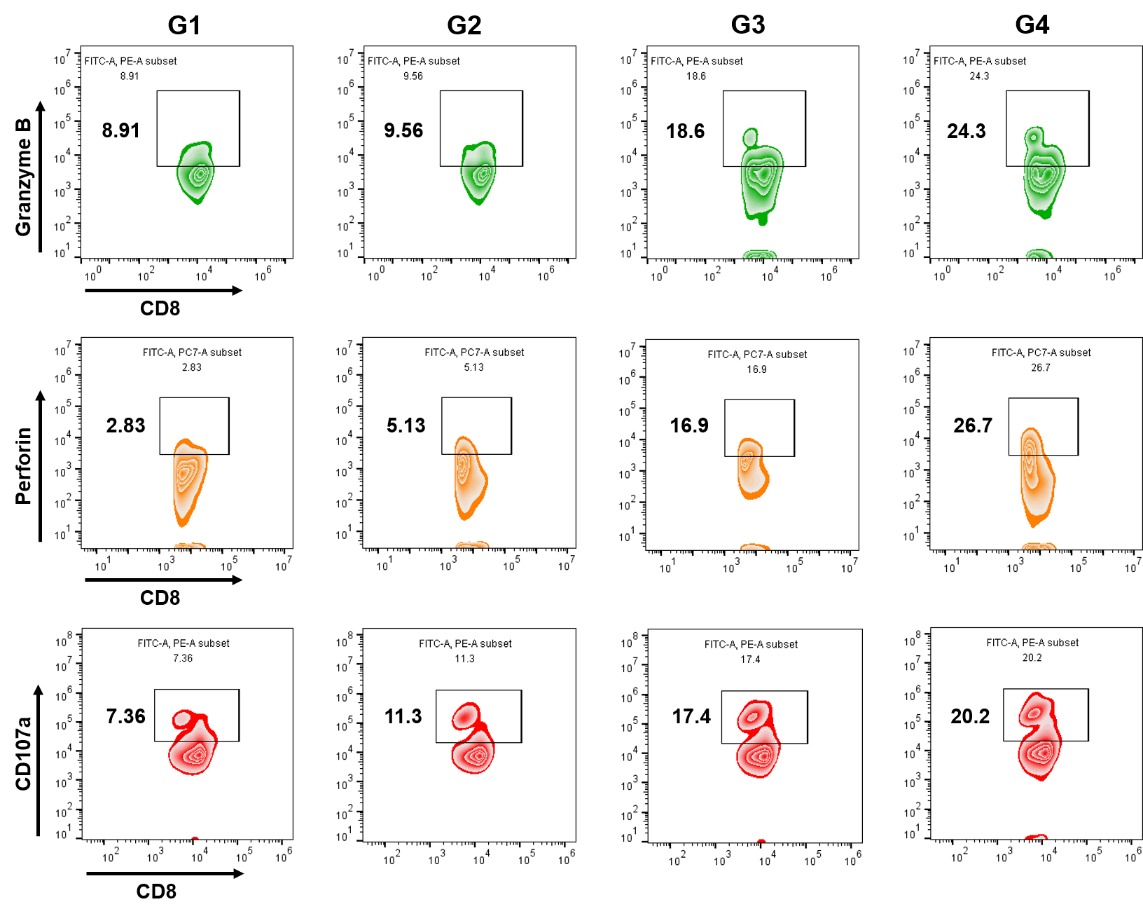
**

**Figure. S16.** The secretion and expression of granzyme B, perforin and CD107a in different treatment groups. G1: PBMC negative control group, G2: PBMC treatment, G3: PBMC+Lip(Met)-R1, G4: PBMC+Lip(MA+Met)-R1.

**
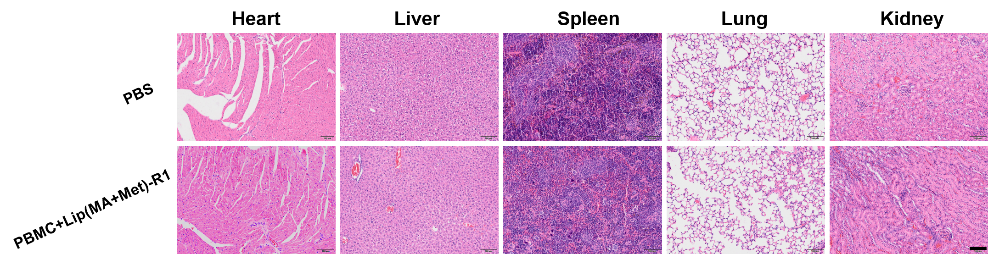
**

**Figure. S17.** H&E staining images of major organs of mice in PBS and PBMC+Lip(MA+Met)-R1 treated groups. Scale bar: 100 μm.

**
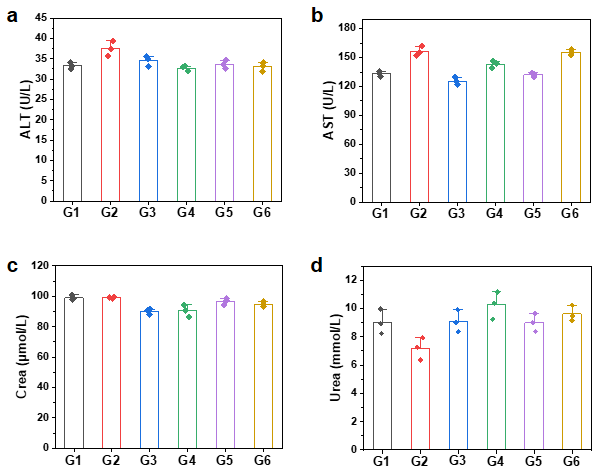
**

**Figure. S18.** Detection of (a) alanine aminotransferase (ALT), (b) aspartate aminotransferase (AST), (c) creatinine (CREA) and (d) urea from mice with different treatments (n = 3 per group). G1: PBS, G2: Lip(MA), G3: Lip(MA)-R1, G4: PBMC, G5: PBMC+Lip(Met)-R1, G6: PBMC+Lip(MA+Met)-R1.

**
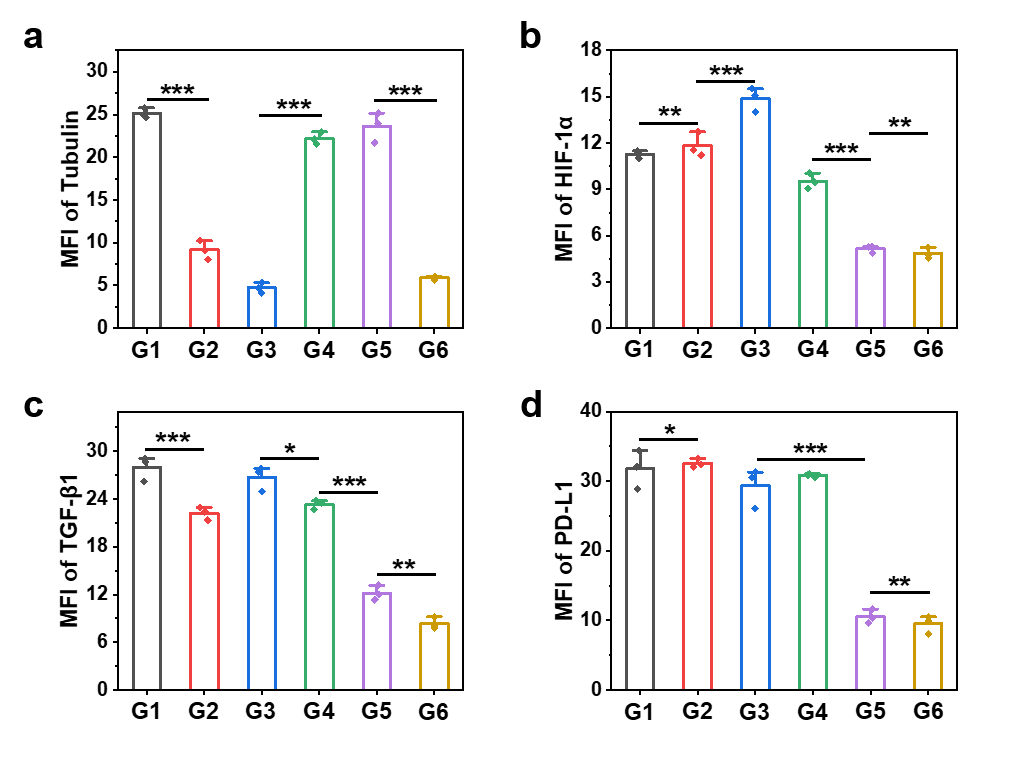
**

**Figure S19.** Quantitative analysis of immunofluorescence staining of tubulin, HIF-1α, TGF-β1 and PD-L1 expression after various treatments. G1: PBS, G2: Lip(MA), G3: Lip(MA)-R1, G4: PBMC, G5: PBMC+Lip(Met)-R1, G6: PBMC+Lip(MA+Met)-R1. **p* < 0.05, ***p* < 0.01 and ****p* < 0.001.

**
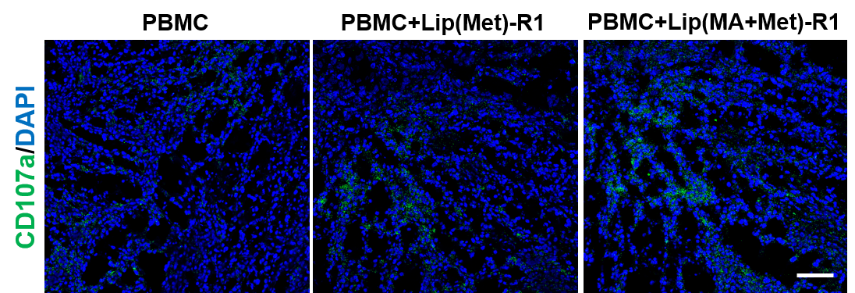
**

**Figure. S20.** Immunofluorescence images of CD107a expression in tumor tissues with different treatments. Scale bar: 50 μm.

**
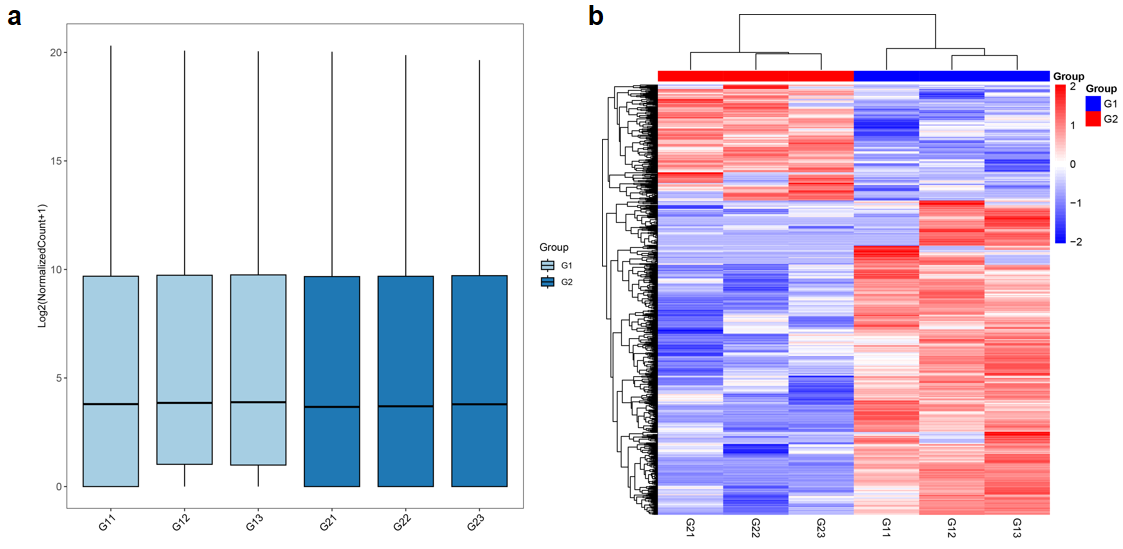
**

**Figure. S21.** (a) Normalization of gene data. (b) Heat map of DEGs in PBMC group (G11, G12, G13) and PBMC+Lip(MA+Met)-R1 group (G21, G22, G23).

**
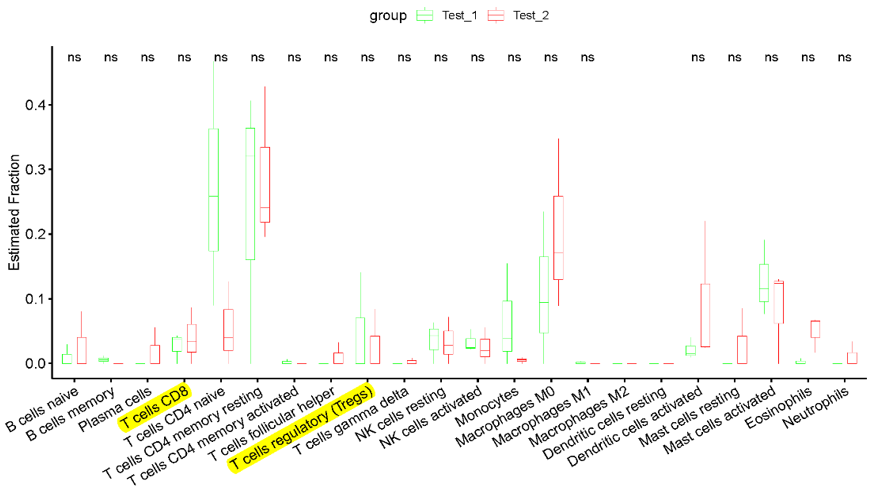
**

**Figure. S22.** The immune infiltration analysis based on RNA-seq data in PBMC group (Test_1) and PBMC+Lip(MA+Met)-R1 group (Test_2).

**
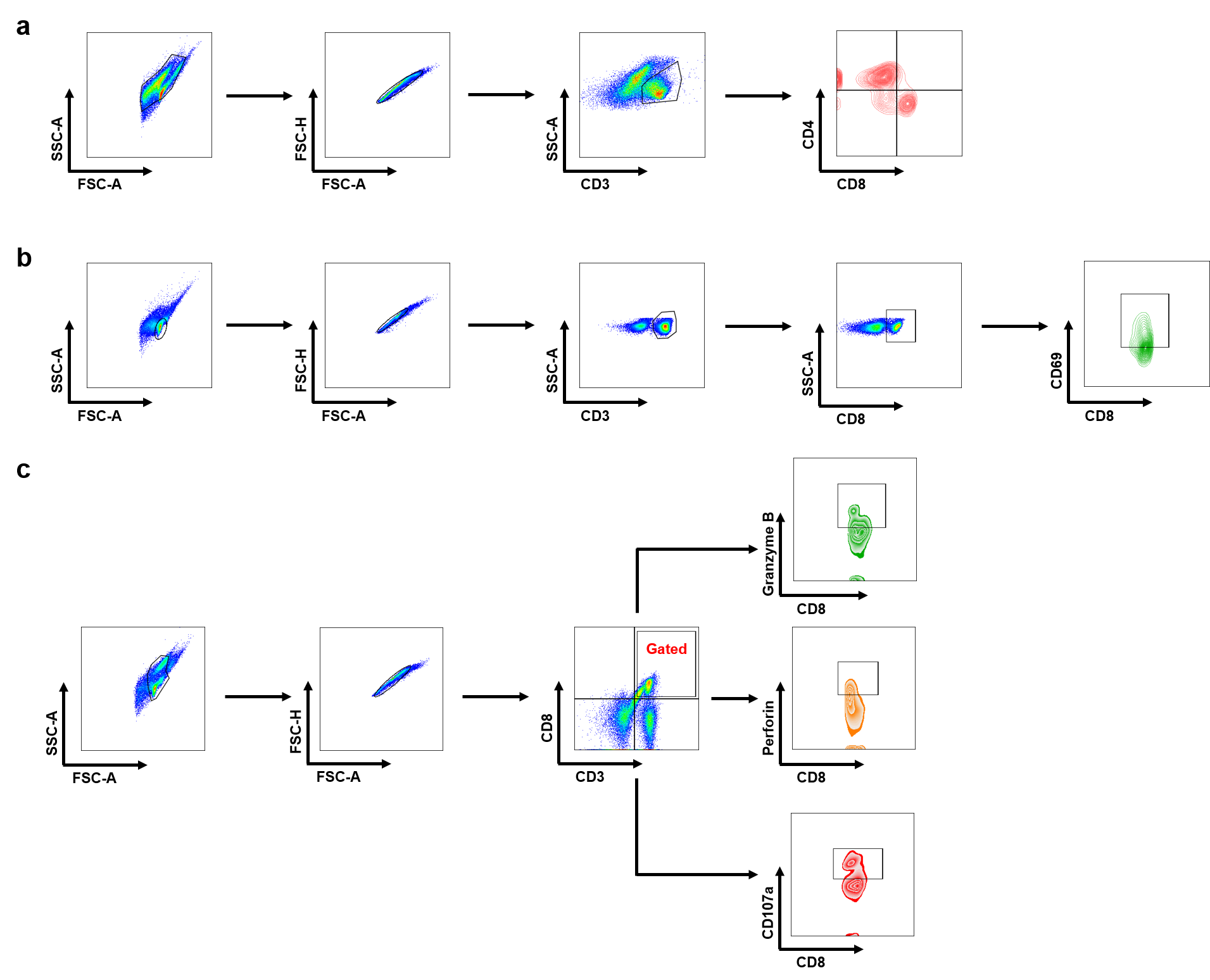
**

**Figure. S23.** The Gating strategy for PMBC mediated immune activation and degranulation effect evaluation.
